# Supplementary material for: Associations of statin adherence and lipid targets with adverse outcomes in myocardial infarction survivors: a retrospective cohort study
Source: BMJ Open. 2021 Sep 27;11(9):e054893. doi: 10.1136/bmjopen-2021-054893 (PMC8477332; doi:10.1136/bmjopen-2021-054893)
Supplement: Supplementary data [file bmjopen-2021-054893supp001.pdf]

BMJ Publishing Group Limited (BMJ) disclaims all liability and responsibility arising from any reliance placed on this supplemental material which has been supplied by the author(s)

Supplementary Material

Table s1: Models for Non-HDL Target by Statin Adherence Thresholds

	Continuous Adherence		50% Adherence		80% Adherence	
**Model/Variable**	**Odds Ratio (95% CI)**	**p-value**	**Odds Ratio (95% CI)**	**p-value**	**Odds Ratio (95% CI)**	**p-value**
Unadjusted Model						
**Adherence Target**						
Achieved (ref.)	--		1.00	<0.0001	1.00	<0.0001
Not Achieved	--		4.77 (3.87, 5.90)		3.03 (2.60, 3.54)	
10% Decrease	1.24 (1.20, 1.27)	<0.0001				
Adjusted Model						
**Adherence Target**						
Achieved (ref.)	--		1.00	<0.0001	1.00	<0.0001
Not Achieved	--		4.47 (3.62, 5.54)		2.98 (2.55, 3.48)	
10% Decrease	1.23 (1.20, 1.26)	<0.0001				

Table s2: Models for LDL Target by Statin Adherence Thresholds

	Continuous Adherence		50% Adherence		80% Adherence	
**Model/Variable**	**Odds Ratio (95% CI)**	**p-value**	**Odds Ratio (95% CI)**	**p-value**	**Odds Ratio (95% CI)**	**p-value**
Unadjusted Model						
**Adherence Target**						
Achieved (ref.)	--		1.00	<0.0001	1.00	<0.0001
Not Achieved	--		1.99 (1.75, 2.26)		1.62 (1.46, 1.80)	
10% Decrease	1.10 (1.08, 1.12)	<0.0001				
Adjusted Model						
**Adherence Target**						
Achieved (ref.)	--		1.00	<0.0001	1.00	<0.0001
Not Achieved	--		2.03 (1.78, 2.31)		1.70 (1.53, 1.88)	
10% Decrease	1.11 (1.09, 1.12)	<0.0001				

Table s3: Models for Non-HDL Target by Statin Adherence Thresholds (sensitivity inc. incomplete years of follow-up)

	Continuous Adherence		50% Adherence		80% Adherence	
**Model/Variable**	**Odds Ratio (95% CI)**	**p-value**	**Odds Ratio (95% CI)**	**p-value**	**Odds Ratio (95% CI)**	**p-value**
Unadjusted Model						
**Adherence Target**						
Achieved (ref.)	--		1.00	<0.0001	1.00	<0.0001
Not Achieved	--		4.30 (3.53, 5.23)		2.85 (2.46, 3.30)	
10% Decrease	1.23 (1.20, 1.26)	<0.0001				
Adjusted Model						
**Adherence Target**						
Achieved (ref.)	--		1.00	<0.0001	1.00	<0.0001
Not Achieved	--		3.98 (3.26, 4.85)		2.75 (2.37, 3.19)	
10% Decrease	1.22 (1.19, 1.25)	<0.0001				

Table s4: Models for LDL Target by Statin Adherence Thresholds (sensitivity inc. incomplete years of follow-up)

	Continuous Adherence		<50% Adherence		<80% Adherence	
**Model/Variable**	**Odds Ratio (95% CI)**	**p-value**	**Odds Ratio (95% CI)**	**p-value**	**Odds Ratio (95% CI)**	**p-value**
Unadjusted Model						
**Adherence Target**						
Achieved (ref.)	--		1.00	<0.0001	1.00	<0.0001
Not Achieved	--		1.86 (1.65, 2.09)		1.53 (1.39, 1.69)	
10% Decrease	1.09 (1.07, 1.10)	<0.0001				
Adjusted Model						
**Adherence Target**						
Achieved (ref.)	--		1.00	<0.0001	1.00	<0.0001
Not Achieved	--		1.90 (1.68, 2.14)		1.59 (1.44, 1.76)	
10% Decrease	1.09 (1.07, 1.11)	<0.0001				

Table s5: Models for All-Cause and CVD Mortality by Lipid Targets

	Non-HDL Target				LDL Target			
	All-Cause		Circulatory		All-Cause		Circulatory	
**Model/Variable**	**Hazard Ratio (95% CI)**	**p-value**	**Hazard Ratio (95% CI)**	**p-value**	**Hazard Ratio (95% CI)**	**p-value**	**Hazard Ratio (95% CI)**	**p-value**
Unadjusted Model								
**Lipid Target**								
Achieved (ref.)	1.00	<0.0001	1.00	<0.0001	1.00	<0.0001	1.00	0.0013
Not Achieved	2.16 (1.87, 2.49)		2.21 (1.72, 2.85)		1.27 (1.16, 1.39)		1.29 (1.11, 1.51)	
Adjusted Model								
**Lipid Target**								
Achieved (ref.)	1.00	<0.0001	1.00	<0.0001	1.00	<0.0001	1.00	0.0012
Not Achieved	1.76 (1.52, 2.03)		1.82 (1.41, 2.34)		1.32 (1.20, 1.44)		1.30 (1.11, 1.52)	

Table s6: Models for Overall Mortality by Statin Adherence Thresholds

	Continuous Adherence		50% Adherence		80% Adherence	
Model/Variable	Hazard Ratio (95% CI)	p-value	Hazard Ratio (95% CI)	p-value	Hazard Ratio (95% CI)	p-value
Unadjusted Model						
**Adherence Target**						
Achieved (ref.)	--		1.00	<0.0001	1.00	<0.0001
Not Achieved	--		1.58 (1.44, 1.73)		1.53 (1.42, 1.66)	
10% Decrease	1.07 (1.06, 1.08)	<0.0001				
Adjusted Model						
**Adherence Target**						
Achieved (ref.)	--		1.00	<0.0001	1.00	<0.0001
Not Achieved	--		1.24 (1.13, 1.37)		1.37 (1.26, 1.48)	
10% Decrease	1.03 (1.02, 1.05)	<0.0001				

Table s7: Models for CVD Mortality by Statin Adherence Thresholds

	Continuous Adherence		50% Adherence		80% Adherence	
Model/Variable	Hazard Ratio (95% CI)	p-value	Hazard Ratio (95% CI)	p-value	Hazard Ratio (95% CI)	p-value
Unadjusted Model						
**Adherence Target**						
Achieved (ref.)	--		1.00	<0.0001	1.00	<0.0001
Not Achieved	--		1.60 (1.36, 1.88)		1.43 (1.25, 1.64)	
10% Decrease	1.06 (1.04, 1.09)	<0.0001				
Adjusted Model						
**Adherence Target**						
Achieved (ref.)	--		1.00	0.0116	1.00	0.0003
Not Achieved	--		1.24 (1.05, 1.46)		1.29 (1.12, 1.48)	
10% Decrease	1.03 (1.01, 1.05)	0.0072				

A – Overall Mortality

	Events	N	Hazard Ratio (95% CI)	p−value
**Average Statin Adherence (Cts)**				
10% Decrease	3748	11031	1.10 (1.09 to 1.11)	<0.0001
	3748	11031	1.06 (1.05 to 1.07)	<0.0001
**Average Statin Adherence (50%)**				
Achieved	2715	8948	1.00	
Not Achieved	1033	2083	2.01 (1.87 to 2.16)	<0.0001
	1033	2083	1.49 (1.38 to 1.60)	<0.0001
**Average Statin Adherence (80%)**				
Achieved	1897	7053	1.00	
Not Achieved	1851	3978	2.05 (1.93 to 2.19)	<0.0001
	1851	3978	1.72 (1.61 to 1.83)	<0.0001
**Non−HDL Target**				
Achieved	286	1709	1.00	
Not Achieved	516	1620	2.18 (1.87 to 2.49)	<0.0001
	516	1620	1.76 (1.52 to 2.03)	<0.0001
**LDL Target**				
Achieved	1886	7171	1.00	
Not Achieved	658	2269	1.27 (1.16 to 1.39)	<0.0001
	658	2269	1.32 (1.20 to 1.44)	<0.0001

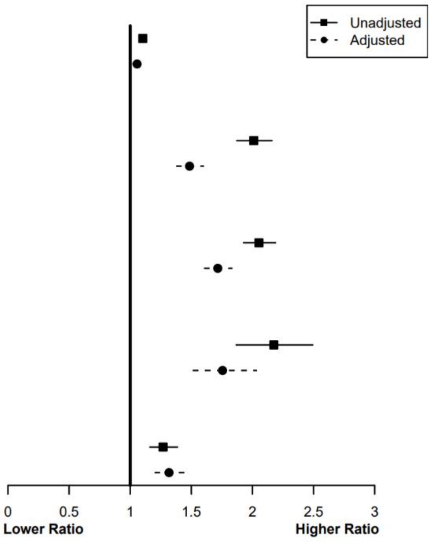

B – CVD Mortality

	Events	N	Hazard Ratio (95% CI)	p−value
**Average Statin Adherence (Cts)**				
10% Decrease	1200	11031	1.08 (1.07 to 1.10)	<0.0001
	1200	11031	1.04 (1.02 to 1.06)	<0.0001
**Average Statin Adherence (50%)**				
Achieved	891	8948	1.00	
Not Achieved	309	2083	1.84 (1.62 to 2.10)	<0.0001
	309	2083	1.35 (1.18 to 1.54)	<0.0001
**Average Statin Adherence (80%)**				
Achieved	646	7053	1.00	
Not Achieved	554	3978	1.81 (1.62 to 2.03)	<0.0001
	554	3978	1.53 (1.37 to 1.72)	<0.0001
**Non−HDL Target**				
Achieved	94	1709	1.00	
Not Achieved	175	1620	2.21 (1.72 to 2.85)	<0.0001
	175	1620	1.82 (1.41 to 2.34)	<0.0001
**LDL Target**				
Achieved	629	7171	1.00	
Not Achieved	222	2269	1.29 (1.11 to 1.51)	0.0013
	222	2269	1.30 (1.11 to 1.52)	0.0012

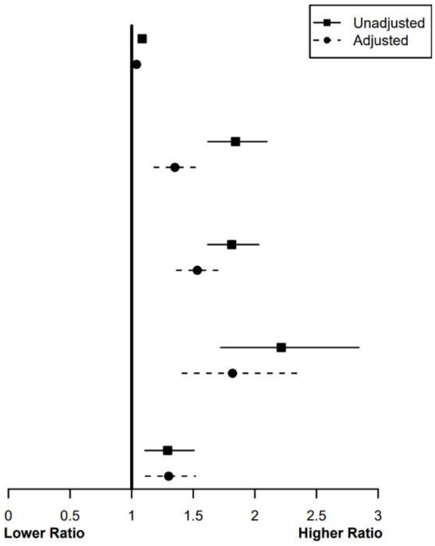

Figure s1: Hazard Ratios of A) All-Cause and B) CVD Mortality by Average Statin Adherence and Achievement of Plasma Lipid Targets (from NICE and ESC Recommendations) (Sensitivity inc. incomplete years of follow up)

Table s8: Models for Overall Mortality by Statin Adherence Thresholds (sensitivity inc. incomplete years of follow up)

	Continuous Adherence		50% Adherence		80% Adherence	
**Model/Variable**	**Hazard Ratio (95% CI)**	**p-value**	**Hazard Ratio (95% CI)**	**p-value**	**Hazard Ratio (95% CI)**	**p-value**
Unadjusted Model						
**Adherence Target**						
Achieved (ref.)	--		1.00	<0.0001	1.00	<0.0001
Not Achieved	--		2.01 (1.87, 2.16)		2.05 (1.93, 2.19)	
10% Decrease	1.10 (1.09, 1.11)	<0.0001				
Adjusted Model						
**Adherence Target**						
Achieved (ref.)	--		1.00	<0.0001	1.00	<0.0001
Not Achieved	--		1.49 (1.38, 1.60)		1.72 (1.61, 1.83)	
10% Decrease	1.06 (1.05, 1.07)	<0.0001				

Table s9: Models for CVD Mortality by Statin Adherence Thresholds (sensitivity inc. incomplete years of follow up)

	Continuous Adherence		50% Adherence		80% Adherence	
**Model/Variable**	**Hazard Ratio (95% CI)**	**p-value**	**Hazard Ratio (95% CI)**	**p-value**	**Hazard Ratio (95% CI)**	**p-value**
Unadjusted Model						
**Adherence Target**						
Achieved (ref.)	--		1.00	<0.0001	1.00	<0.0001
Not Achieved	--		1.84 (1.62, 2.10)		1.81 (1.62, 2.03)	
10% Decrease	1.09 (1.07, 1.10)	<0.0001				
Adjusted Model						
**Adherence Target**						
Achieved (ref.)	--		1.00	0.0001	1.00	<0.0001
Not Achieved	--		1.35 (1.18, 1.54)		1.53 (1.37, 1.72)	
10% Decrease	1.04 (1.02, 1.06)	<0.0001				

Table s10: Models for Recurrent MIs by Lipid Targets

	Non-HDL Target		LDL Target	
**Model/Variable**	**Odds Ratio (95% CI)**	**p-value**	**Odds Ratio (95% CI)**	**p-value**
Unadjusted Model				
**Lipid Target**				
Achieved (ref.)	1.00	0.0127	1.00	0.8776
Not Achieved	1.21 (1.04, 1.40)		1.01 (0.91, 1.12)	
Adjusted Model				
**Lipid Target**				
Achieved (ref.)	1.00	0.0161	1.00	0.9419
Not Achieved	1.20 (1.03, 1.40)		1.00 (0.90, 1.12)	

Table s11: Models for Recurrent MIs by Statin Adherence Thresholds

	Continuous Adherence		50% Adherence		80% Adherence	
**Model/Variable**	**Odds Ratio (95% CI)**	**p-value**	**Odds Ratio (95% CI)**	**p-value**	**Odds Ratio (95% CI)**	**p-value**
Unadjusted Model						
**Adherence Target**						
Achieved (ref.)	--		1.00	0.1221	1.00	0.1309
Not Achieved	--		0.91 (0.81, 1.03)		1.07 (0.98, 1.18)	
10% Decrease	1.00 (0.98, 1.01)	0.5141				
Adjusted Model						
**Adherence Target**						
Achieved (ref.)	--		1.00	0.0803	1.00	0.1346
Not Achieved	--		0.90 (0.79, 1.01)		1.07 (0.98, 1.18)	
10% Decrease	0.99 (0.98, 1.01)	0.4278				

	Events	N	Odds Ratio (95% CI)	p−value
**Average Statin Adherence (Cts)**				
10% Decrease	3265	11031	0.99 (0.98 to 1.01)	0.3392
	3265	11031	0.99 (0.98 to 1.00)	0.2279
**Average Statin Adherence (50%)**				
Achieved	2684	8948	1.00	
Not Achieved	581	2083	0.90 (0.81 to 1.00)	0.0572
	581	2083	0.89 (0.80 to 0.99)	0.0255
**Average Statin Adherence (80%)**				
Achieved	2049	7053	1.00	
Not Achieved	1216	3978	1.08 (0.99 to 1.17)	0.1305
	1216	3978	1.07 (0.98 to 1.16)	0.1305
**Non−HDL Target**				
Achieved	490	1709	1.00	
Not Achieved	529	1620	1.21 (1.04 to 1.40)	0.0127
	529	1620	1.20 (1.03 to 1.40)	0.0161
**LDL Target**				
Achieved	2159	7171	1.00	
Not Achieved	687	2269	1.01 (0.91 to 1.12)	0.8776
	687	2269	1.00 (0.90 to 1.12)	0.9419

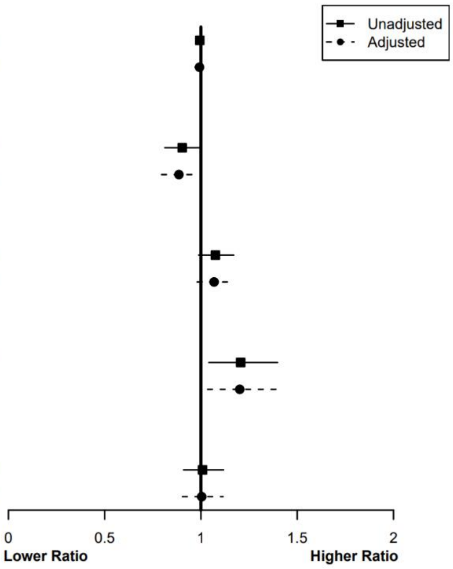

Figure s2: Odds of MIs During Follow Up by Average Statin Adherence and Achievement of Plasma Lipid Targets (from NICE and ESC Recommendations) (Sensitivity inc. incomplete years of follow up)

Table s12: Models for Recurrent MIs by Average Statin Adherence Thresholds (sensitivity inc. incomplete years)

	Continuous Adherence		50% Adherence		80% Adherence	
**Model/Variable**	**Odds Ratio (95% CI)**	**p-value**	**Odds Ratio (95% CI)**	**p-value**	**Odds Ratio (95% CI)**	**p-value**
Unadjusted Model						
**Adherence Target**						
Achieved (ref.)	--		1.00	0.0572	1.00	0.0943
Not Achieved	--		0.90 (0.81, 1.00)		1.08 (0.99, 1.17)	
10% Decrease	0.99 (0.98, 1.01)	0.3392				
Adjusted Model						
**Adherence Target**						
Achieved (ref.)	--		1.00	0.0255	1.00	0.1305
Not Achieved	--		0.89 (0.80, 0.99)		1.07 (0.98, 1.16)	
10% Decrease	1.00 (0.98, 1.00)	0.2279				
