# Supplementary material for: Associations of statin adherence and lipid targets with adverse outcomes in myocardial infarction survivors: a retrospective cohort study
Source: BMJ Open. 2021 Sep 27;11(9):e054893. doi: 10.1136/bmjopen-2021-054893 (PMC8477332; doi:10.1136/bmjopen-2021-054893)
Supplement: Author's manuscript [file bmjopen-2021-054893.draft_revisions.pdf]

BMJ Open

BMJ Open is committed to open peer review. As part of this commitment we make the peer review history of every article we publish publicly available.

When an article is published we post the peer reviewers' comments and the authors' responses online. We also post the versions of the paper that were used during peer review. These are the versions that the peer review comments apply to.

The versions of the paper that follow are the versions that were submitted during the peer review process. They are not the versions of record or the final published versions. They should not be cited or distributed as the published version of this manuscript.

BMJ Open is an open access journal and the full, final, typeset and author-corrected version of record of the manuscript is available on our site with no access controls, subscription charges or pay-per-view fees (http://bmjopen.bmj.com).

If you have any questions on BMJ Open's open peer review process please email info.bmjopen@bmj.com

BMJ Open

Associations of statin adherence and lipid targets with adverse outcomes in myocardial infarction survivors: a retrospective cohort study

Journal:	*BMJ Open*
Manuscript ID	bmjopen-2021-054893
Article Type:	Original research
Date Submitted by the Author:	29-Jun-2021
Complete List of Authors:	Brown, Rosemary; University of Glasgow, Institute of Cardiovascular and Medical Sciences<br>Lewsey, Jim; University of Glasgow<br>Wild, Sarah; University of Edinburgh, Public Health Sciences<br>Logue, Jennifer; Lancaster University, Lancaster Medical School<br>Welsh, Paul; University of Glasgow, Institute of Cardiovascular and Medical Sciences; University Of Glasgow
Keywords:	Cardiac Epidemiology < CARDIOLOGY, Myocardial infarction < CARDIOLOGY, EPIDEMIOLOGY

SCHOLARONE™
Manuscripts

I, the Submitting Author has the right to grant and does grant on behalf of all authors of the Work (as defined in the below author licence), an exclusive licence and/or a non-exclusive licence for contributions from authors who are: i) UK Crown employees; ii) where BMJ has agreed a CC-BY licence shall apply, and/or iii) in accordance with the terms applicable for US Federal Government officers or employees acting as part of their official duties; on a worldwide, perpetual, irrevocable, royalty-free basis to BMJ Publishing Group Ltd ("BMJ") its licensees and where the relevant Journal is co-owned by BMJ to the co-owners of the Journal, to publish the Work in this journal and any other BMJ products and to exploit all rights, as set out in our licence.

The Submitting Author accepts and understands that any supply made under these terms is made by BMJ to the Submitting Author unless you are acting as an employee on behalf of your employer or a postgraduate student of an affiliated institution which is paying any applicable article publishing charge ("APC") for Open Access articles. Where the Submitting Author wishes to make the Work available on an Open Access basis (and intends to pay the relevant APC), the terms of reuse of such Open Access shall be governed by a Creative Commons licence – details of these licences and which Creative Commons licence will apply to this Work are set out in our licence referred to above.

Other than as permitted in any relevant BMJ Author's Self Archiving Policies, I confirm this Work has not been accepted for publication elsewhere, is not being considered for publication elsewhere and does not duplicate material already published. I confirm all authors consent to publication of this Work and authorise the granting of this licence.

For peer review only

Associations of statin adherence and lipid targets with adverse outcomes in myocardial infarction survivors: a retrospective cohort study

Authors:

Rosemary Brown[a], Jim Lewsey[b], Sarah Wild[c], Jennifer Logue *[d], Paul Welsh*[a]

*Joint senior authors

[a]BHF Glasgow Cardiovascular Research Centre, Institute of Cardiovascular and Medical Science, University of Glasgow, Glasgow, UK.

[b]Institute of Health & Wellbeing, University of Glasgow, Glasgow, UK.

[c]Usher Institute, University of Edinburgh, Edinburgh, UK.

[d]Lancaster Medical School, Health Innovation One, Sir John Fisher Drive, Lancaster University, Lancaster, UK.

Corresponding Author:

Paul Welsh

Institute of Cardiovascular and Medical Sciences, University of Glasgow, BHF Glasgow Cardiovascular Research Centre, 126 University Place, Glasgow G12 8TA, UK.

Tel: +44 (0)141 330 1722

Email address: paul.welsh@glasgow.ac.uk

Word Count (Main Body): 3,438

Number of Tables and Figures: 4

I, the Submitting Author has the right to grant and does grant on behalf of all authors of the Work (as defined in the below author licence), an exclusive licence and/or a non-exclusive licence for contributions from authors who are: i) UK Crown employees; ii) where BMJ has agreed a CC-BY licence shall apply, and/or iii) in accordance with the terms applicable for US Federal Government officers or employees acting as part of their official duties; on a worldwide, perpetual, irrevocable, royalty-free basis to BMJ Publishing Group Ltd ("BMJ") its licensees and where the relevant Journal is co-owned by BMJ to the co-owners of the Journal, to publish the Work in BMJ Open and any other BMJ products and to exploit all rights, as set out in our licence.

For peer review only

For peer review only - http://bmjopen.bmj.com/site/about/guidelines.xhtml

Abstract

Objectives To examine associations between statin adherence and lipid target achievement in myocardial infarction (MI) survivors, and their associations with recurrent MIs and mortality.

Design Retrospective cohort study using linked clinical records within the National Health Service Greater Glasgow and Clyde (NHS GGC) Data Safe Haven.

Setting Routine clinical practice in the NHS GGC area between January 2009 and July 2017.

Participants Patients ≥18 years who experienced a non-fatal MI hospital admission (ICD10: I21, I22) between January 2009 and July 2014 (n=11,031), followed up from the date of MI admission until July 2017 or death, whichever occurred first.

Primary and Secondary Outcome Measures Statin adherence was estimated using encashed prescriptions, and lipid results from routine biochemistry data. Primary lipid and statin adherence targets were LDL≤1.8mmol/L and adherence ≥50%, and were related to all-cause death, deaths due to cardiovascular disease (CVD) (ICD10: I00-I99 as the underlying cause), and recurrent MI in unadjusted models and models adjusting for age, sex, socioeconomic deprivation, and year of MI.

Results Over 4.5 years follow-up, 76% achieved LDL≤1.8mmol/L, and 84.5% had average adherence ≥50%. Patients with adherence <50% had an increased risk of not meeting LDL≤1.8mmol/L, in adjusted models (OR (95% CI): 2.03 (1.78, 2.31), p<0.0001). In univariable models, not meeting LDL≤1.8mmol/L was associated with increased risks of all-cause mortality (HR 1.27 (1.16, 1.39), p<0.0001) and CVD mortality (1.29 (1.11, 1.51), p=0.0013). Adherence <50% was associated with increased risks of all-cause mortality (1.58 (1.44, 1.74), p<0.0001) and CVD mortality (1.60 (1.36, 1.88), p<0.0001). Adjustment for confounders did not abrogate these associations. Neither exposure was associated with recurrent MIs.

Conclusions Non-achievement of lipid and adherence targets are similarly associated with increased risks of all-cause and CVD mortality. Further work is required to optimise their use to improve outcomes in clinical practice.

Abstract Word Count: 294/300

For peer review only - http://bmjopen.bmj.com/site/about/guidelines.xhtml

Strengths and limitations of this study

- Data were derived from routine clinical practice records within a large health board in the West of Scotland, with a reasonable average duration of follow up (4.5 years).
- The use of encashed prescriptions in estimating adherence may be stronger evidence of medicines use over issued prescriptions.
- In mortality analysis, time-window bias could have arisen as patients with events had reduced opportunities to achieve lipid targets or improve average statin adherence.
- Suitable baseline lipid results were substantially missing within secondary prevention populations (~70%), limiting the utility of percentage change targets in this analysis.

For peer review only

Introduction

Those with established CVD (i.e., secondary prevention) are universally identified and targeted in risk management strategies due to their markedly higher rates of further cardiovascular events and overall mortality [1,2]. A cornerstone of these strategies is lipid-lowering medications, where statins are the first-line therapy.

Adherence to these medications and attaining lipid targets are key components of a patient review to address their cardiovascular risk in Scotland and the rest of the UK [3,4]. Indeed, achievement of target lipid levels (set by clinical guidelines) has been associated with a reduced likelihood of further events and mortality [1,5]. The association between adherence and the risk of cardiovascular events has also been shown in primary and secondary prevention in observational data, although adherence was measured using prescriptions issued rather than by drugs dispensed [6,7].

There is evidence to suggest adherence is higher in secondary prevention than primary prevention [8], and analyses have found reasonable adherence in secondary prevention cohorts; between 68% and 75% had statin adherence ≥80% in the year following an event [9,10]. However, this was not translated into target lipid levels, where fewer than half of the patients achieved these in the same period [10]. Despite this, many of the risk factors associated with CVD have also been shown to be associated with adherence and achievement of target lipid levels. For example, men [11,12], and, in primary prevention, those with a diagnosis of diabetes and hypertension [12] are more likely to be adherent. In secondary prevention, however, insulin use has been associated with non-adherence [13], although in a separate secondary prevention cohort, those with a diabetes diagnosis were more likely to meet lipid targets [10]. Therefore, there superficially appears to be differences between lipid-lowering medication adherence and lipid target achievement. This leads to uncertainties in clinical guidelines and in clinical practice as to whether adherence or lipid targets are more useful surrogates of risk reduction [14].

This analysis, using routinely collected data available for a secondary prevention population representative of a UK health board region, seeks to investigate associations between statin adherence and lipid target achievement, and their associations with recurrent MIs and mortality. This analysis also investigates which is more strongly associated with outcomes, and therefore relevant for clinical decision making.

For peer review only - http://bmjopen.bmj.com/site/about/guidelines.xhtml

Methods

Data

The data used for this analysis was a subset of an extract of all individuals in NHS Greater Glasgow (GGC) and Clyde, a UK National Health Service region in the West of Scotland, who had a lipid profile result or a prescription for a statin, ezetimibe, or PCSK9 inhibitor before 29th December 2017. This initial data extraction was performed by staff in the NHS GGC Data Safe Haven and contained data for 652,441 individuals (Project Reference: GSH/17/CA/012 PMCVD; NHS GGC Safe Haven approval covers governance and ethical approvals). Data extracted included demographics, laboratory results, dispensed prescriptions, hospital admissions, death certificates, and diabetes diagnoses.

To maximise completeness, patients were followed up between 1st January 2009 and 31st July 2017 (inclusive). Patients who resided outside the region, were <18 years, died before 1st January 2009, had multiple death certificates, or received apparent posthumous lipid tests or prescriptions for lipid-lowering medications more than six months posthumously were excluded. Figure 1 provides an overview of the cohort derivation.

Patient and Public Involvement

Neither patients nor the public were involved in the design, conduct, reporting, or dissemination of this research.

Post MI Population

MIs were identified in linked hospital admission records (ICD10: I21, I22 in any diagnosis field). To allow for at least three years follow-up (although death may occur in these years), patients who did not have an MI between 1st January 2009 and 31st July 2014 were excluded. Patients who only experienced fatal MIs (death occurred during admission or within 30 days of discharge), whose statin adherence was >200%, or were missing a socioeconomic deprivation index were also removed. Patients were followed up from their first non-fatal MI admission until 31st July 2017 or death, whichever occurred first. Year-long time windows were constructed for each patient, starting from their baseline admission date.

Statin Adherence

Statin prescription dispensing records were used to estimate patients' adherence. Dispensed dates were used as this was the first date that the patient could have possession of the medication.

For peer review only - http://bmjopen.bmj.com/site/about/guidelines.xhtml

Prescription end dates were calculated as the dispensed date plus the day-coverage dispensed, and quantities were adjusted to reflect dose availability where overlaps across time windows or death occurred. The Medication Possession Ratio (MPR) [15] was calculated as (number of doses dispensed in time window/length of time window)*100 for each full-year time window, and mean-averaged. Average adherence was considered continuously and dichotomously, using cut-offs at 50% [16] and 80% [15,17], with 50% the primary exposure of interest.

Lipid Targets

Lipid profiles were collected as part of routine clinical practice in NHS Biochemistry laboratories. A lipid profile result consisted of five components: Total Cholesterol, Total Cholesterol:HDL Cholesterol, HDL Cholesterol, LDL Cholesterol, and Triglycerides. If a patient had multiple tests within a time window, the mean value of the tests was calculated. Non-HDL cholesterol was calculated as total cholesterol minus HDL cholesterol.

The primary lipid exposure of interest was the 2016 European Society of Cardiology (ESC) target for LDL cholesterol ≤1.8mmol/l [18], and the 2014 National Institute for Health and Care Excellence (NICE) target for a ≥40% reduction in non-HDL cholesterol [3] was a secondary lipid exposure of interest. The latter, though not formally recommended as a target, is also referred to within current Scottish guidelines, where achievement is considered indicative of adequate adherence to statin therapy [4]. To assess the attainment of the non-HDL target, a pre-MI statin-naïve baseline was required and was identified as the last lipid profile test a patient had before their baseline admission that was more than six months (182 days) after the end date of any previous statin prescription. The need for a baseline result limited the coverage of this exposure to 30%.

Outcomes

Underlying cause and date of death were obtained from linked death certificates and hospital discharge notices, with CVD deaths defined as ICD10: I00-I99. Patients missing a cause of death were assumed to have a non-CVD underlying cause of death. All MI events from linked hospital admission records (Scottish Morbidity Record (SMR01)) and death certificates before the end of follow-up were included as a separate outcome (ICD-10: I21, I22).

Other Covariates

Date of birth and sex was extracted from clinical records. For socioeconomic deprivation, the Scottish Index of Multiple Deprivation (SIMD) was obtained from the patients' residential postcode

For peer review only - http://bmjopen.bmj.com/site/about/guidelines.xhtml

at baseline. Due to the higher number of the most deprived zones in this region than in Scotland in general [19], NHS GGC specific SIMD quintiles were derived to effectively assess the impact of deprivation within the post MI population of NHS GGC. The patient's age at MI was calculated using their date of birth and date of baseline MI. Prior MIs were defined as MI admissions occurring in the ten years before their baseline. The date of diagnosis and the type of diabetes was obtained from routine electronic diabetes records. The date of diagnosis was taken as the patient's first valid (i.e. after the patent's date of birth) date of entry in the diabetes register. Patients were considered to have had a diagnosis of diabetes at the time of their MI if this date was before their baseline MI admission. The type of diabetes was extracted, and if multiple types were listed during a patient's history, the most common diagnosis was taken.

Statistical Analysis

All analyses were performed using R v3.5.0 [20], and the 'forestplot' [21], 'survival' [22], and 'survminer' [23] packages. Continuous variables were summarised using mean and standard deviation and categorical variables with the number of observations (N) and percentages. As a patient's average adherence was calculated using complete years of follow-up, only patients who survived ≥1 year were included in analyses including adherence. For analyses including lipid targets, patients were included if they reported ≥1 test result during follow-up and were classified as meeting a target if they achieved it at any stage during follow-up.

Logistic regression was used to understand the associations between average adherence and the achievement of lipid targets, and the association between adherence, and lipid targets, with recurrent MIs. For mortality, Cox regression was conducted, and the proportional hazards assumption was checked by visual inspection of the partial Schoenfeld residuals. For all outcomes, unadjusted and adjusted (for age at MI, sex, deprivation quintile, and year of MI) models were generated, using available case analysis for each exposure. Where adherence was considered dichotomously, and for the lipid targets, achievement of the threshold or target was the reference. A sensitivity analysis for adherence was also conducted by including events that occurred in the first year of follow-up. For all analyses, a p-value <0.05 was considered statistically significant.

Results

Baseline Demographics

Of the 11,031 patients, 10,009 (90.7%) had an average adherence calculable, 3,329 (30.2%) had ≥1 percentage change in non-HDL cholesterol result, and 9,440 (85.6%) had ≥1 LDL cholesterol result.

For peer review only - http://bmjopen.bmj.com/site/about/guidelines.xhtml

Over an average 4.5 years follow-up, 85% achieved ≥50% average statin adherence and 69% had ≥80%. Three-quarters of patients had LDL≤1.8mmol/L and half achieved ≥40% reduction in non-HDL at least once during follow-up.

Table 1 shows demographics by lipid target achievement and average adherence. Males were more likely to be adherent and achieve lipid targets. Patients who achieved ≥40% reduction in non-HDL and patients who were adherent (at either threshold) were younger on average. Those residing in more deprived areas were more likely to have adherence ≥50%, but this was less evident for ≥80%, and those in the most and least deprived areas were more likely to meet the LDL target. Patients with a diagnosis of diabetes were more likely to achieve LDL≤1.8mmol/l but were less likely to meet the non-HDL target. However, patients diagnosed with Type 2 diabetes were more likely to be adherent, particularly ≥50%, whilst patients diagnosed with Type 1 diabetes were less likely. Finally, patients with prior MIs were slightly more likely to achieve LDL≤1.8mmol/L and have adherence ≥50%, but the inverse was true for the non-HDL target.

Adherence and Lipid Targets

Adherence was associated with lipid target achievement (Tables s1-2). Those with adherence <50% had an increased risk of not meeting LDL≤1.8mmol/L in unadjusted (OR 1.99 (95% CI 1.75, 2.26), p<0.0001) and adjusted models (OR 2.03 (1.78, 2.31), p<0.0001), and had an adjusted odds ratio of not meeting the non-HDL target of 4.5 (95% CI: 3.62, 5.54, p<0.0001). Similar patterns were observed for adherence <80%. A 10% decrease in adherence was associated with a 20% increase in odds of not meeting the non-HDL target, and a 10% increase in odds of not achieving LDL≤1.8mmol/L, with both increases unaltered following adjustment. In a sensitivity analysis including incomplete years of follow-up, odds ratios were slightly lower but retained statistical significance (Tables s3-4).

Associations with All-Cause and Cardiovascular Mortality

All adherence and lipid target exposures were associated with all-cause and CVD mortality. For all-cause mortality in unadjusted models, non-achievement of LDL≤1.8mmol/L and the non-HDL target was associated with 1.3 times and 2.2 times greater risks, respectively (Figure 2a, Table s5). Following adjustment, this risk remained similar for LDL≤1.8mmol/L but attenuated to 1.8 times higher for the non-HDL target. The hazard ratios were nearly identical for CVD mortality, although fewer events resulted in wider confidence intervals (Figure 2b, Table s5).

For peer review only - http://bmjopen.bmj.com/site/about/guidelines.xhtml

For average adherence, similar effect sizes were reported for both all-cause and CVD mortality (Figure 2, Tables s6-7), with. In unadjusted analyses, adherence <50% was associated with a hazard of all-cause mortality 1.6 times, and adherence <80% with a hazard 1.5 times, that of those above each threshold. A 10% decrease in adherence was associated with 7% and 6% increases in the risk of all-cause and circulatory mortality respectively, which reduced to 3% in adjusted analyses. For the 50% and 80% thresholds, adjustment attenuated the hazard ratios to 1.2 and 1.4, respectively. In a sensitivity analysis including incomplete years of follow-up in the average adherence calculation, the unadjusted hazard ratios were slightly larger, increasing to two times higher risk for all-cause mortality, and 1.8 times higher for CVD mortality (Figure s1, Tables s8-9). When adjusted, this attenuated to hazard ratios of 1.5 for <50% and 1.7 for <80% adherence for all-cause mortality, and 1.4 and 1.5 for CVD mortality for <50% and <80% adherence respectively.

Associations with Recurrent Myocardial Infarction

In both unadjusted and adjusted models, there were no associations between the achievement of LDL≤1.8mmol/l, or statin adherence, and recurrent MIs during follow-up (Figure 3, Tables s10-11). Nevertheless, there was an association with the non-HDL target; non-achievement was associated with 21% higher odds of recurrent MI in unadjusted analyses. For all models, adjustment had minimal impact on the odds ratios. In a sensitivity analysis (Figure s2, Table s12), where average adherence included incomplete years, these associations remained consistent.

Discussion

In this Scottish secondary prevention population, 76% achieved LDL≤1.8mmol/L, 51% achieved ≥40% reduction in non-HDL, and 85% and 69% had an average statin adherence ≥50% and ≥80% respectively. Lower average statin adherence, and failure to achieve guideline-recommended lipid targets, were strongly associated with higher risks of all-cause and CVD mortality and were significantly associated with each other. In adjusted models, those with adherence <50% were 24% more likely to die, and those not achieving LDL≤1.8mmol/L were 32% more likely to die, with similar patterns observed when cardiovascular causes were considered.

This study advances the literature by the simultaneous comparison of both adherence and lipid targets as surrogates of statin effectiveness, showing these measurements have similar associations with outcomes. However, guidelines do not currently recommend the use of adherence data in estimating the clinical effectiveness of statins in patients. Our data support the notion that a patient's statin adherence, estimated using MPR, may be a useful risk surrogate, and that a 50%

For peer review only - http://bmjopen.bmj.com/site/about/guidelines.xhtml

threshold appears to have approximately similar associations with outcomes as an 80% threshold. Given that alternate-day statin dosing is a common approach in routine clinical practice where daily statins are not tolerated [16], the 50% threshold, therefore, seems pragmatic. Furthermore, these data also suggest that where adherence data are readily available, annual lipid tests may not be necessary for CVD risk assessment in secondary prevention, potentially reducing costs and workload for health services. However, this hypothesis requires further research.

Whilst on-statin lipid targets are somewhat controversial [24,25], and there is a lack of robust evidence to support their use in guidelines [14], lowering LDL has consistently demonstrated significant reductions in mortality within those with established disease [1]. Consistent with this, we report associations of LDL and non-HDL targets with all-cause and CVD specific mortality outcomes. The stronger associations with the non-HDL target than the associations with the LDL target are likely to have arisen due to the nature of the targets e.g. percentage change vs absolute value, rather than the lipid profile components themselves. For example, associations between absolute values of non-HDL and LDL with a composite endpoint of cardiovascular events are approximately equivalent [26]. Nevertheless, percentage change targets within secondary prevention could prove problematic, with many patients unlikely to suitable baselines for the calculation to be performed. This was evident here where percentage change could only be calculated for 30% of the cohort.

The consistent associations we report between adherence and mortality are also broadly similar to findings observed in other cohorts [1,7]. In high-risk patients in a UK primary care cohort, adherence <80% was associated with a 50% greater hazard of all cardiovascular events following adjustment for demographic factors and comorbidities [7]. Our data suggest that a 50% adherence threshold had a similar association in unadjusted models, although this was attenuated after adjustment.

There were no significant associations between average statin adherence nor the achievement of lipid targets with recurrent MIs. This second observation is consistent with other observational cohorts where post MI LDL demonstrated poor predictive performance for recurrent ASCVD events [27,28]. However, statin non-adherence has previously demonstrated a stronger, and intensity-dependent, association with further events in a cohort study in Finland between 2004 and 2016 (n=28,625) [28]. One explanation for this could be that in our analysis, average statin adherence and lipid target achievement was determined from information collected before and after recurrent MIs, whereas Lassenius et al (2020) used only data for the intervening time [28]. Furthermore, as many patients in our analysis experienced MIs early in follow-up, these exposures are more likely to reflect

For peer review only - http://bmjopen.bmj.com/site/about/guidelines.xhtml

patient behaviour following multiple MIs, rather than the intervening time. For example, patients may initially increase their adherence or treatment intensity or only achieve lipid targets, after further MIs [10,28]. However, one analysis found only 5% more patients achieved LDL≤1.8mmol/L and 7% increased their treatment intensity after their second event [10].

The associations between statin adherence and lipid target achievement also expand on associations with mortality, with similar associations reported in other observational cohorts using electronic prescribing records. For example, in one analysis in Georgia, USA (n=~1,000), those with adherence <50%, as measured using proportion of days covered from dispensing data, were at 88% greater risk of not achieving a 30% reduction in LDL [11]. However, in a separate analysis of UK primary care data utilising prescribing data, following a cardiovascular event, approximately 70% of patients achieved ≥80% statin adherence (as measured by proportion of days covered) in the first year, despite a higher percentage failing to have LDL≤1.8mmol/l [10]. Therefore, whilst adherence appears to be associated with cholesterol levels, it is not the only contributing factor. This pattern is also observed in this cohort, which calculated MPR, a similar and widely used method, on dispensing data.

Strengths and Limitations

This is a contemporary, large dataset from a real-world population. The generalisability of these findings, particularly to the UK population, is therefore good. In this analysis, we confirm and expand on previous research by illustrating that lipid levels and statin adherence are important predictors of death and increase the duration of follow-up beyond the initial few years following statin initiation. This facilitated the estimation of longer-term average statin adherence and more time for target lipid levels to be achieved. Furthermore, the adherence measure used is arguably stronger evidence of medication use due to prescriptions being encashed [29], rather than prescribed.

In models using average adherence, only those who survived ≥1 year of follow-up were included, reducing the potential for reverse causality. Indeed, in sensitivity analyses, effect sizes were larger for mortality and smaller for lipid target achievement where incomplete time windows were included. However, in all mortality models, time-window bias could have arisen due to differing lengths of follow-up [30]. Specifically, patients who died had reduced opportunities to achieve lipid targets and change their adherence behaviour.

Finally, adjustment for age, sex, deprivation, and year of MI, did not substantially alter the effect sizes or their significance. However, this adjustment was not comprehensive, and other confounders

For peer review only - http://bmjopen.bmj.com/site/about/guidelines.xhtml

For peer review only

are likely to remain. For example, adherence in secondary prevention is higher in those with a greater number of comorbidities [10,11], which may also increase the risk of further events. In this cohort, those experiencing further events were more likely to have a diagnosis of diabetes or had a prior MI; both of which have been associated with higher adherence [10,12]. Consequently, given the observational nature of these data, no causal conclusions are drawn.

Conclusions

A significant proportion of the post MI population achieves lipid targets and high average statin adherence, and both have similar strengths of association with reduced mortality. These associations, and those observed between lipid target achievement and adherence, are consistent with the evidence base for statins. We also broadly validate the existing Scottish SIGN non-HDL guidance as clinically relevant, however low coverage for percentage change variables in secondary prevention limits its practical use in these settings.

Funding

RB is funded as part of the Medical Research Council Doctoral Training Programme in Precision Medicine (MR/N013166/1). The Medical Research Council has had no role in any aspect of this work.

Competing Interests

RB, JLewsey, SW, and JLogue declare no conflict. PW has received grant support from Roche Diagnostics, AstraZeneca, Novartis, and Boehringer Ingelheim.

Author's Contributions

JLogue and PW contributed to the conception and design of the work. All authors contributed to the acquisition, analysis and interpretation. RB drafted the manuscript, and all authors critically revised it. All gave final approval and agreed to be accountable for all aspects of work ensuring integrity and accuracy.

Ethical Approval Information

This study used fully anonymised health record data accessed via a trusted research environment and was therefore exempt from ethical review. Permission was given by NHS GGC Safe Haven local Privacy Advisory Committee (membership includes the ethics committee chair) (project GSH/17/CA/012 PMCVD).

For peer review only - http://bmjopen.bmj.com/site/about/guidelines.xhtml

For peer review only

Data Availability Statement

Deidentified data may be obtained by bona fide researchers, for the purposes of research in the public interest, from NHS GGC Safe Haven, via an approval process (https://www.nhsggc.org.uk/about-us/professional-support-sites/glasgow-safe-haven/). Code files are available on request (Rosemary.Brown@glasgow.ac.uk).

For peer review only

For peer review only - http://bmjopen.bmj.com/site/about/guidelines.xhtml

References

1 Cholesterol Treatment Trialists' (CTT) Collaboration. Efficacy and safety of more intensive lowering of LDL cholesterol: a meta-analysis of data from 170 000 participants in 26 randomised trials. *Lancet* 2010;**376**:1670–81. doi:10.1016/S0140-6736(10)61350-5

2 World Health Organization. Cardiovascular Diseases (CVDs): Fact Sheet. 2017.https://www.who.int/en/news-room/fact-sheets/detail/cardiovascular-diseases-(cvds) (accessed 3 Oct 2019).

3 NICE. Cardiovascular disease: risk assessment and reduction, including lipid modification (CG181). 2014.

4 SIGN. SIGN 149: Risk estimation and the prevention of cardiovascular disease. Published Online First: 2017.http://www.sign.ac.uk/assets/sign149.pdf

5 Cholesterol Treatment Trialists' (CTT) Collaborators. The effects of lowering LDL cholesterol with statin therapy in people at low risk of vascular disease: meta-analysis of individual data from 27 randomised trials. *Lancet* 2012;**380**:581–90. doi:10.1016/S0140-6736(12)60367-5

6 Herrett E, Gallagher AM, Bhaskaran K, *et al.* Data Resource Profile: Clinical Practice Research Datalink (CPRD). *Int J Epidemiol* 2015;**44**:827–36. doi:10.1093/ije/dyv098

7 Khunti K, Danese MD, Kutikova L, *et al.* Association of a Combined Measure of Adherence and Treatment Intensity With Cardiovascular Outcomes in Patients With Atherosclerosis or Other Cardiovascular Risk Factors Treated With Statins and/or Ezetimibe. *JAMA Netw Open* 2018;**1**. doi:10.1001/jamanetworkopen.2018.5554

8 Naderi SH, Bestwick JP, Wald DS. Adherence to drugs that prevent cardiovascular disease: Meta-analysis on 376,162 patients. *Am J Med* 2012;**125**:882-887.e1. doi:10.1016/j.amjmed.2011.12.013

9 Nordstrom BL, Collins JM, Donaldson R, *et al.* Treatment patterns and lipid levels among patients with high-risk atherosclerotic CVD in the UK. *Br J Cardiol* Published Online First: 2015. doi:10.5837/bjc.2015.034

10 Danese MD, Gleeson M, Kutikova L, *et al.* Management of lipid-lowering therapy in patients with cardiovascular events in the UK: A retrospective cohort study. *BMJ Open* 2017;**7**:1–10. doi:10.1136/bmjopen-2016-013851

11 Vupputuri S, Joski PJ, Kilpatrick R, *et al.* LDL cholesterol response and statin adherence among high-risk patients initiating treatment. *J Manag Care* 2016;**22**.https://www.ajmc.com/journals/issue/2016/2016-vol22-n3/ldl-cholesterol-response-and-statin-adherence-among-high-risk-patients-initiating-treatment

12 Hope HF, Binkley GM, Fenton S, *et al.* Systematic review of the predictors of statin adherence for the primary prevention of cardiovascular disease. *PLoS One* 2019;**14**. doi:10.1371/journal.pone.0201196

13 Chen ST, Huang ST, Shau WY, *et al.* Long-term statin adherence in patients after hospital discharge for new onset of atherosclerotic cardiovascular disease: A population-based study of real world prescriptions in Taiwan. *BMC Cardiovasc Disord* 2019;**19**. doi:10.1186/s12872-019-1032-4

14 Brown RE, Welsh P, Logue J. Systematic review of clinical guidelines for lipid lowering in the secondary prevention of cardiovascular disease events. *Open Hear* 2020;**7**:e001396. doi:10.1136/openhrt-2020-001396

15 Raebel MA, Schmittdiel J, Karter AJ, *et al.* Standardizing Terminology and Definitions of Medication Adherence and Persistence in Research employing Electronic Databases. *Med Care* 2014;**51**:11–21. doi:10.1097/MLR.0b013e31829b1d2a.Standardizing

16 Jordanov MS, Abou Assi H. Statins. In: Yassine H, ed. *Lipid Management: From Basics to Clinic*. Springer International Publishing 2015. 123–47. doi:10.1007/978-3-319-11161-2_8

17 Karve S, Cleves MA, Helm M, *et al.* Good and poor adherence: optimal cut-point for adherence measures using administrative claims data. *Curr Med Res Opin* 2009;**25**:2303–10. doi:10.1185/03007990903126833

For peer review only - http://bmjopen.bmj.com/site/about/guidelines.xhtml

18 Piepoli MF, Hoes AW, Agewall S, *et al.* 2016 European Guidelines on cardiovascular disease prevention in clinical practice. *Eur Heart J* 2016;**37**:2315–81. doi:10.1093/eurheartj/ehw106
19 NHS Greater Glasgow and Clyde. NHS GGC Director of Public Health Report 2015-2017; Chapter 5. 2015. https://www.nhsggc.org.uk/media/234515/nhsggc_ph_dphreport2015_health_related_behaviours.pdf
20 R Core Team. R: A Language and Environment for Statistical Computing. 2018.http://www.r-project.org/
21 Gordon M, Lumley T. forestplot: Advanced Forest Plot Using 'grid' Graphics in R. 2020.https://cran.r-project.org/web/packages/forestplot/index.html (accessed 15 Jan 2021).
22 Therneau TM. A Package for Survival Analysis in R. 2020.https://cran.r-project.org/package=survival
23 Kassambara A, Kosinski M, Biecek P, *et al.* Drawing Survival Curves using 'ggplot2'. 2020.https://cran.r-project.org/package=survminer
24 Leibowitz M, Cohen-Stavi C, Basu S, *et al.* Targeting LDL Cholesterol: Beyond Absolute Goals Toward Personalized Risk. *Curr Cardiol Rep* 2017;**19**:52. doi:10.1007/s11886-017-0858-6
25 Pallazola V, Cardoso R, Blumenthal RS, *et al.* Was the Juice Worth the Squeeze? Understanding the New 2018 AHA/ACC Cholesterol Guideline - American College of Cardiology. Am. Coll. Cardiol. Latest Cardiol. 2018.https://www.acc.org/latest-in-cardiology/articles/2018/11/14/10/48/was-the-juice-worth-the-squeeze (accessed 25 Mar 2020).
26 Welsh C, Celis-Morales CA, Brown R, *et al.* Comparison of Conventional Lipoprotein Tests and Apolipoproteins in the Prediction of Cardiovascular Disease: Data from UK Biobank. *Circulation* Published Online First: 2019. doi:10.1161/CIRCULATIONAHA.119.041149
27 Ohm J, Hjemdahl P, Skoglund PH, *et al.* Lipid levels achieved after a first myocardial infarction and the prediction of recurrent atherosclerotic cardiovascular disease. *Int J Cardiol* 2019;**296**:1–7. doi:10.1016/j.ijcard.2019.07.001
28 Lassenius MI, Toppila I, Bergius S, *et al.* Cardiovascular event rates increase after each recurrence and associate with poor statin adherence. *Eur J Prev Cardiol* 2020;:2047487320904334. doi:10.1177/2047487320904334
29 Information Services Division. Prescribing Information System Fields Document. Prescr. Inf. Syst. Fields (1993 - Present. 2010;:18.https://www.isdscotland.org/Health-Topics/Prescribing-and-Medicines/Prescribing-Datamarts/docs/PIS_fields_for_researchers_v5_eDRIS Guidance.pdf (accessed 3 May 2018).
30 Di Martino M, Kirchmayer U, Agabiti N, *et al.* The impact of time-window bias on the assessment of the long-term effect of medication adherence: The case of secondary prevention after myocardial infarction. *BMJ Open* 2015;**5**:e007866. doi:10.1136/bmjopen-2015-007866

For peer review only - http://bmjopen.bmj.com/site/about/guidelines.xhtml

Figure Legends

Figure 1: Derivation of the Post Myocardial Infarction Population; MI = Myocardial Infarction, SIMD = Scottish Index of Multiple Deprivation.

Figure 2: Hazard Ratios of A) All-Cause and B) CVD Mortality by Average Statin Adherence and Achievement of Plasma Lipid Targets. Ratios presented for unadjusted (solid line and square) and adjusted (dashed line and circle) analyses (adjusted for age, sex, year of MI, and SIMD quintile). CVD death defined by ICD10 I00-I99 as the underlying cause. Average adherence calculated as the patient's mean-average annual medication possession ratio of complete years of follow-up. Non-HDL target: ≥40% reduction from a pre-MI baseline, LDL target: ≤1.8mmol/l. Patients classified as meeting a plasma lipid target if they achieved the target in any year of their follow-up, and not meeting a target otherwise. Cts = Continuous; CI = Confidence Interval.

Figure 3: Odds of MIs During Follow-Up by Average Statin Adherence and Achievement of Plasma Lipid Targets (from NICE and ESC Recommendations). Ratios presented for unadjusted (solid line and square) and adjusted (dashed line and circle) analyses (adjusted for age, sex, year of MI, and SIMD quintile). CVD death defined by ICD10 I00-I99 as the underlying cause. Models fitted using available case analysis. Average adherence calculated as the patient's mean-average annual medication possession ratio of complete years of follow-up. Non-HDL target: ≥40% reduction from a pre-MI baseline, LDL target: ≤1.8mmol/l. Patients classified as meeting a plasma lipid target if they achieved the target in any year of their follow-up, and not meeting a target otherwise. Cts = Continuous; CI = Confidence Interval.

For peer review only

For peer review only - http://bmjopen.bmj.com/site/about/guidelines.xhtml

Table 1: Demographics by Average Statin Adherence (50% and 80%) and Achievement of Lipid Targets

Target	Average Adherence ≥50%		Average Adherence ≥80%		≥40% reduction non-HDL*		LDL ≤1.8mmol/l	
	Met<br>8,461 (84.5%)	Not Met<br>1,548 (15.5%)	Met<br>6,894 (68.9%)	Not Met<br>3,115 (31.1%)	Met<br>1,709 (51.3%)	Not Met<br>1,620 (48.7%)	Met<br>7,171 (76.0%)	Not Met<br>2,269 (24.0%)
**Gender**								
Male	5,337 (63.1%)	829 (53.6%)	4,368 (63.4%)	1,798 (57.7%)	1,102 (64.5%)	911 (56.2%)	4,566 (63.7%)	1,342 (59.1%)
Female	3,124 (36.9%)	719 (46.4%)	2,526 (36.6%)	1,317 (42.3%)	607 (35.5%)	709 (43.8%)	2,605 (36.3%)	927 (40.9%)
**Age at MI (years)**								
Mean (SD)	65.3 (13.2)	68.1 (15.9)	65.4 (13.0)	66.4 (15.2)	63.6 (12.6)	67.0 (13.5)	65.4 (13.2)	65.5 (13.9)
**SIMD 2012 Quintile (NHS GGC)**								
1 (Most)	2,058 (24.3%)	347 (22.4%)	1,652 (24.0%)	753 (24.2%)	398 (23.3%)	381 (23.5%)	1,824 (25.4%)	412 (18.2%)
2	1,979 (23.4%)	318 (20.5%)	1,625 (23.6%)	672 (21.6%)	389 (22.8%)	363 (22.4%)	1,620 (22.6%)	551 (24.3%)
3	1,750 (20.7%)	338 (21.8%)	1,389 (20.1%)	699 (22.4%)	349 (20.4%)	349 (21.5%)	1,438 (20.1%)	535 (23.6%)
4	1,395 (16.5%)	289 (18.7%)	1,153 (16.7%)	531 (17.0%)	284 (16.6%)	283 (17.5%)	1,140 (15.9%)	454 (20.0%)
5 (Least)	1,279 (15.1%)	256 (16.5%)	1,075 (15.6%)	460 (14.8%)	289 (16.9%)	244 (15.1%)	1,149 (16.0%)	317 (14.0%)
**Diabetes at MI**	1,535 (18.1%)	258 (16.7%)	1,249 (18.1%)	544 (17.5%)	285 (16.7%)	369 (22.8%)	1,436 (20.0%)	312 (13.8%)
Type 1	80 (0.9%)	26 (1.7%)	63 (0.9%)	43 (1.4%)	12 (0.7%)	29 (1.8%)	73 (1.0%)	32 (1.4%)
Type 2	1,455 (17.2%)	232 (15.0%)	1,186 (17.2%)	501 (16.1%)	273 (16.0%)	340 (21.0%)	1,363 (19.0%)	280 (12.3%)
**Prior MI**	529 (6.3%)	85 (5.5%)	425 (6.2%)	189 (6.1%)	34 (2.0%)	98 (6.0%)	471 (6.6%)	114 (5.0%)
1	412 (4.9%)	68 (4.4%)	330 (4.8%)	150 (4.8%)	28 (1.6%)	79 (4.9%)	359 (5.0%)	94 (4.1%)
>1	117 (1.4%)	17 (1.1%)	95 (1.4%)	39 (1.3%)	6 (0.4%)	19 (1.2%)	112 (1.6%)	20 (0.9%)

***refers to the percentage reduction in non-HDL cholesterol from the pre-MI baseline. MI = Myocardial Infarction; SD = Standard Deviation; IQR = Interquartile Range, SIMD = Scottish Index of Multiple Deprivation. Numbers are N (%) unless otherwise specified. Percentages are calculated within columns, except for the header where percentages are calculated from total with information. For adherence, patients were only included if ≥1 complete year of follow-up was available. For lipid targets, patients were included if a necessary test result was available.**

For peer review only - http://bmjopen.bmj.com/site/about/guidelines.xhtml

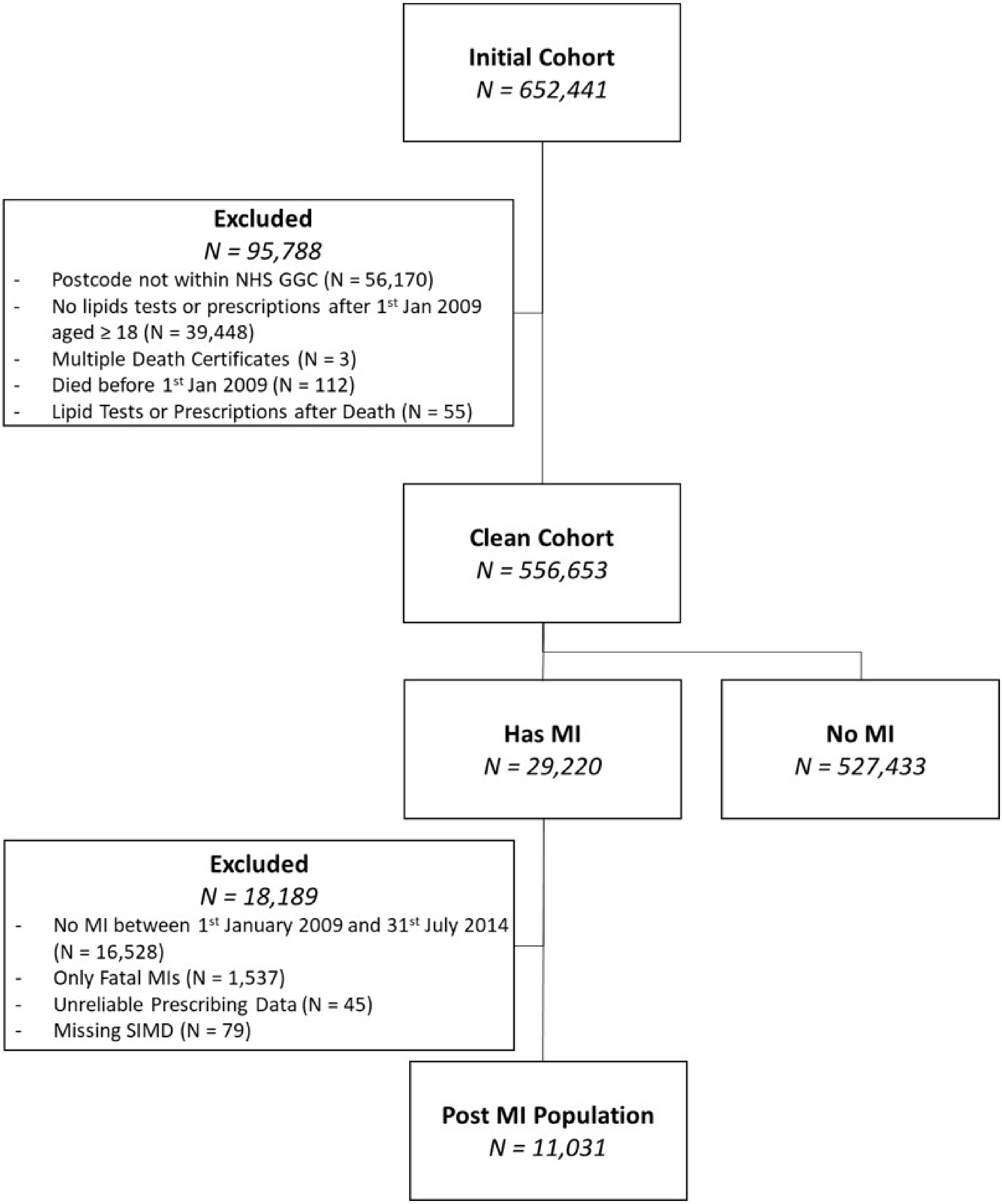
Initial Cohort
N = 652,441
Excluded
N = 95,788
- Postcode not within NHS GGC (N = 56,170)
- No lipids tests or prescriptions after 1st Jan 2009 aged ≥ 18 (N = 39,448)
- Multiple Death Certificates (N = 3)
- Died before 1st Jan 2009 (N = 112)
- Lipid Tests or Prescriptions after Death (N = 55)
Clean Cohort
N = 556,653
Has MI
N = 29,220
No MI
N = 527,433
Excluded
N = 18,189
- No MI between 1st January 2009 and 31st July 2014 (N = 16,528)
- Only Fatal MIs (N = 1,537)
- Unreliable Prescribing Data (N = 45)
- Missing SIMD (N = 79)
Post MI Population
N = 11,031

A – Overall Mortality

	Events	N	Hazard Ratio (95% CI)	p-value
**Average Statin Adherence (Cts)**				
10% Decrease	2726	10009	1.07 (1.06 to 1.08)	<0.0001
	2726	10009	1.03 (1.02 to 1.05)	<0.0001
**Average Statin Adherence (50%)**				
Achieved	2162	8461	1.00	
Not Achieved	564	1548	1.58 (1.44 to 1.74)	<0.0001
	564	1548	1.24 (1.13 to 1.37)	<0.0001
**Average Statin Adherence (80%)**				
Achieved	1656	6894	1.00	
Not Achieved	1070	3115	1.53 (1.42 to 1.66)	<0.0001
	1070	3115	1.37 (1.26 to 1.48)	<0.0001
**Non−HDL Target**				
Achieved	286	1709	1.00	
Not Achieved	516	1620	2.18 (1.87 to 2.49)	<0.0001
	516	1620	1.76 (1.52 to 2.03)	<0.0001
**LDL Target**				
Achieved	1886	7171	1.00	
Not Achieved	658	2269	1.27 (1.16 to 1.39)	<0.0001
	658	2269	1.32 (1.20 to 1.44)	<0.0001

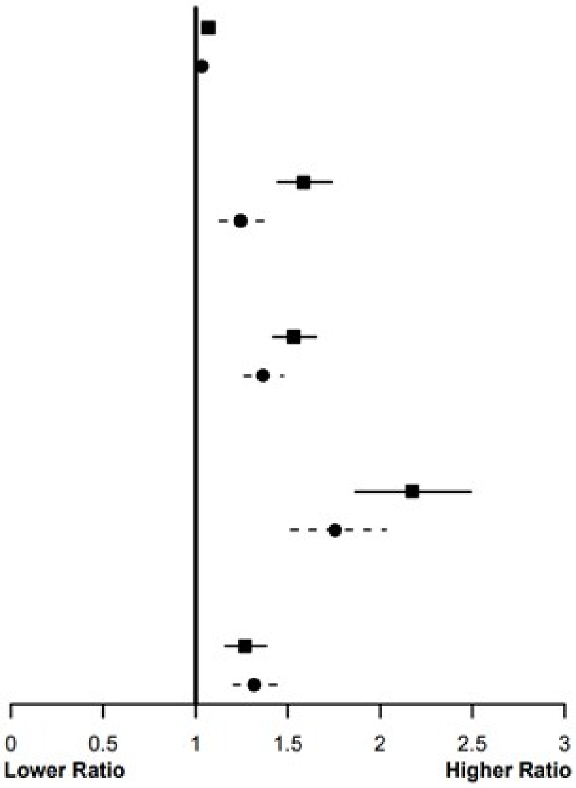

B – CVD Mortality

	Events	N	Hazard Ratio (95% CI)	p-value
**Average Statin Adherence (Cts)**				
10% Decrease	894	10009	1.06 (1.04 to 1.09)	<0.0001
	894	10009	1.03 (1.01 to 1.05)	0.0072
**Average Statin Adherence (50%)**				
Achieved	708	8461	1.00	
Not Achieved	186	1548	1.60 (1.36 to 1.88)	<0.0001
	186	1548	1.24 (1.05 to 1.46)	0.0116
**Average Statin Adherence (80%)**				
Achieved	558	6894	1.00	
Not Achieved	336	3115	1.43 (1.25 to 1.64)	<0.0001
	336	3115	1.29 (1.12 to 1.48)	0.0003
**Non−HDL Target**				
Achieved	94	1709	1.00	
Not Achieved	175	1620	2.21 (1.72 to 2.85)	<0.0001
	175	1620	1.82 (1.41 to 2.34)	<0.0001
**LDL Target**				
Achieved	629	7171	1.00	
Not Achieved	222	2269	1.29 (1.11 to 1.51)	0.0013
	222	2269	1.30 (1.11 to 1.52)	0.0012

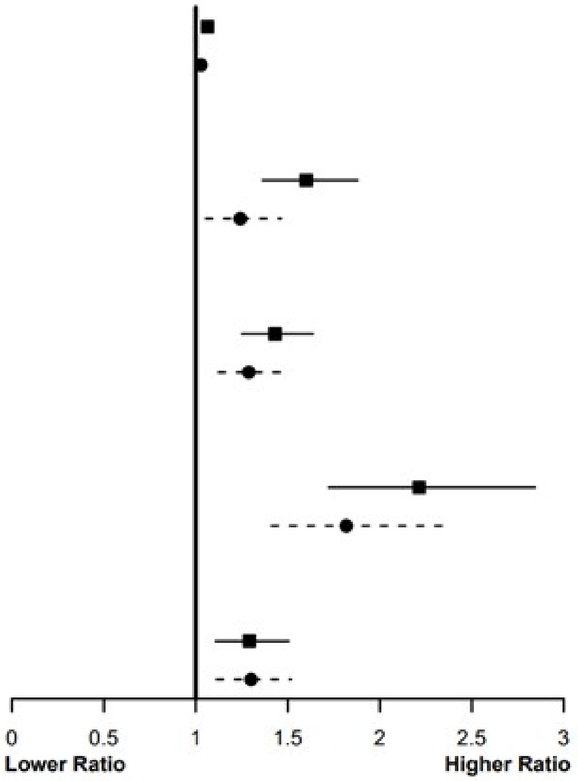

	Events	N	Odds Ratio (95% CI)	p−value
**Average Statin Adherence (Cts)**				
10% Decrease	2918	10009	1.00 (0.98 to 1.01)	0.5141
	2918	10009	0.99 (0.98 to 1.01)	0.4278
**Average Statin Adherence (50%)**				
Achieved	2492	8461	1.00	
Not Achieved	426	1548	0.91 (0.81 to 1.03)	0.1221
	426	1548	0.90 (0.79 to 1.01)	0.0803
**Average Statin Adherence (80%)**				
Achieved	1978	6894	1.00	
Not Achieved	940	3115	1.07 (0.98 to 1.18)	0.1309
	940	3115	1.07 (0.98 to 1.18)	0.1346
**Non−HDL Target**				
Achieved	490	1709	1.00	
Not Achieved	529	1620	1.21 (1.04 to 1.40)	0.0127
	529	1620	1.20 (1.03 to 1.40)	0.0161
**LDL Target**				
Achieved	2159	7171	1.00	
Not Achieved	687	2269	1.01 (0.91 to 1.12)	0.8776
	687	2269	1.00 (0.90 to 1.12)	0.9419

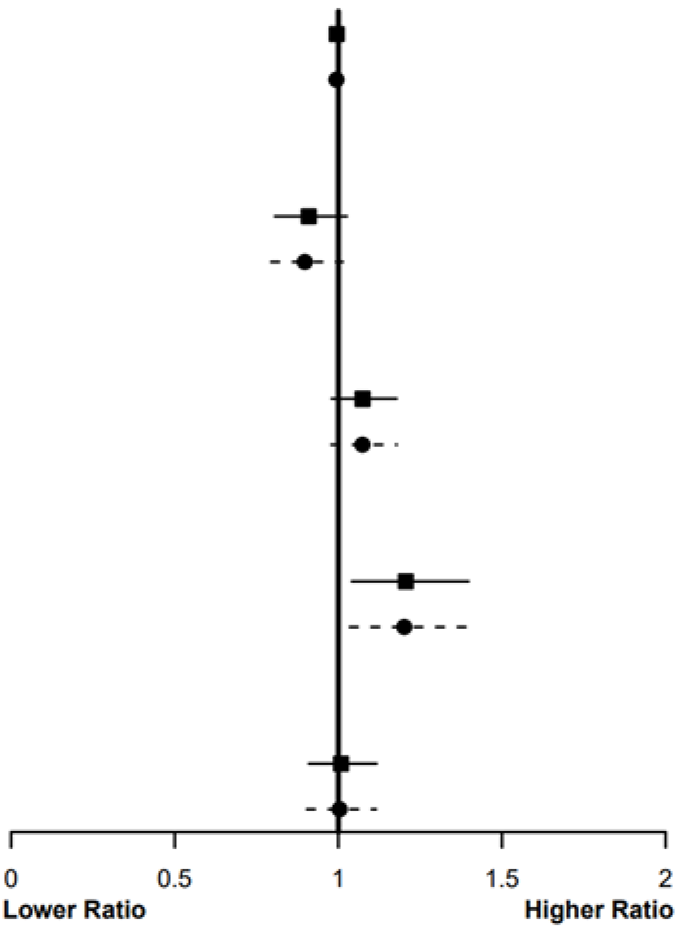

Supplementary Material

For peer review only

Table s1: Models for Non-HDL Target by Statin Adherence Thresholds

	Continuous Adherence		50% Adherence		80% Adherence	
**Model/Variable**	**Odds Ratio (95% CI)**	**p-value**	**Odds Ratio (95% CI)**	**p-value**	**Odds Ratio (95% CI)**	**p-value**
Unadjusted Model						
**Adherence Target**						
Achieved (ref.)	--		1.00	<0.0001	1.00	<0.0001
Not Achieved	--		4.77 (3.87, 5.90)		3.03 (2.60, 3.54)	
10% Decrease	1.24 (1.20, 1.27)	<0.0001				
Adjusted Model						
**Adherence Target**						
Achieved (ref.)	--		1.00	<0.0001	1.00	<0.0001
Not Achieved	--		4.47 (3.62, 5.54)		2.98 (2.55, 3.48)	
10% Decrease	1.23 (1.20, 1.26)	<0.0001				
**Sex**						
Female (ref.)	1.00	0.0062	1.00	0.0046	1.00	0.0014
Male	0.81 (0.69, 0.94)		0.80 (0.69, 0.93)		0.78 (0.67, 0.91)	
**Age at MI (years)**	1.01 (1.01, 1.02)	<0.0001	1.01 (1.01, 1.02)	<0.0001	1.02 (1.01, 1.02)	<0.0001
**SIMD (NHS GGC)**						
1 [most]	1.34 (1.05, 1.71)	0.2136	1.32 (1.04, 1.68)	0.2405	1.26 (0.99, 1.60)	0.3969
2	1.24 (0.97, 1.58)		1.24 (0.97, 1.58)		1.22 (0.96, 1.55)	
3	1.21 (0.94, 1.54)		1.23 (0.96, 1.56)		1.17 (0.92, 1.49)	
4	1.22 (0.95, 1.58)		1.21 (0.94, 1.57)		1.21 (0.94, 1.56)	
5 [least] (ref.)	1.00		1.00		1.00	
**Year of MI**						
2009 (ref.)	1.00	0.9033	1.00	0.9455	1.00	0.7523
2010	1.12 (0.82, 1.54)		1.13 (0.82, 1.54)		1.19 (0.87, 1.62)	
2011	1.12 (0.83, 1.51)		1.12 (0.83, 1.51)		1.14 (0.85, 1.54)	
2012	1.19 (0.88, 1.60)		1.18 (0.88, 1.58)		1.25 (0.93, 1.67)	
2013	1.16 (0.86, 1.56)		1.13 (0.84, 1.51)		1.23 (0.91, 1.65)	
2014	1.18 (0.85, 1.65)		1.12 (0.81, 1.55)		1.23 (0.89, 1.70)	

For peer review only

Table s2: Models for LDL Target by Statin Adherence Thresholds

Model/Variable	Continuous Adherence Odds Ratio (95% CI)	Continuous Adherence p-value	50% Adherence Odds Ratio (95% CI)	50% Adherence p-value	80% Adherence Odds Ratio (95% CI)	80% Adherence p-value
Unadjusted Model						
**Adherence Target**						
Achieved (ref.)	--		1.00	<0.0001	1.00	<0.0001
Not Achieved	--		1.99 (1.75, 2.26)		1.62 (1.46, 1.80)	
10% Decrease	1.10 (1.08, 1.12)	<0.0001				
Adjusted Model						
**Adherence Target**						
Achieved (ref.)	--		1.00	<0.0001	1.00	<0.0001
Not Achieved	--		2.03 (1.78, 2.31)		1.70 (1.53, 1.88)	
10% Decrease	1.11 (1.09, 1.12)	<0.0001				
**Sex**						
Female (ref.)	1.00	0.0002	1.00	<0.0001	1.00	<0.0001
Male	0.82 (0.73, 0.91)		0.81 (0.73, 0.90)		0.80 (0.72, 0.89)	
**Age at MI (years)**	1.00 (0.99, 1.00)	0.0285	1.00 (0.99, 1.00)	0.0306	1.00 (0.99, 1.00)	0.0757
**SIMD (NHS GGC)**						
1 [most]	0.81 (0.68, 0.97)	<0.0001	0.81 (0.68, 0.97)	<0.0001	0.79 (0.67, 0.95)	<0.0001
2	1.23 (1.04, 1.45)		1.22 (1.04, 1.45)		1.21 (1.02, 1.42)	
3	1.28 (1.09, 1.52)		1.29 (1.09, 1.53)		1.27 (1.07, 1.50)	
4	1.52 (1.28, 1.81)		1.51 (1.27, 1.80)		1.51 (1.27, 1.79)	
5 [least] (ref.)	1.00		1.00		1.00	
**Year of MI**						
2009 (ref.)	1.00	<0.0001	1.00	<0.0001	1.00	<0.0001
2010	1.11 (0.92, 1.34)		1.11 (0.92, 1.33)		1.12 (0.93, 1.35)	
2011	1.39 (1.16, 1.66)		1.38 (1.15, 1.65)		1.40 (1.17, 1.68)	
2012	1.40 (1.17, 1.68)		1.39 (1.16, 1.66)		1.43 (1.19, 1.71)	
2013	1.67 (1.39, 1.99)		1.63 (1.37, 1.96)		1.70 (1.42, 2.03)	
2014	4.14 (3.41, 5.02)		4.05 (3.34, 4.91)		4.16 (3.43, 5.05)	

For peer review only

Table s3: Models for Non-HDL Target by Statin Adherence Thresholds (sensitivity inc. incomplete years of follow-up)

Model/Variable	Continuous Adherence		50% Adherence		80% Adherence	
	Odds Ratio (95% CI)	p-value	Odds Ratio (95% CI)	p-value	Odds Ratio (95% CI)	p-value
Unadjusted Model						
**Adherence Target**						
Achieved (ref.)	--		1.00	<0.0001	1.00	<0.0001
Not Achieved	--		4.30 (3.53, 5.23)		2.85 (2.46, 3.30)	
10% Decrease	1.23 (1.20, 1.26)	<0.0001				
Adjusted Model						
**Adherence Target**						
Achieved (ref.)	--		1.00	<0.0001	1.00	<0.0001
Not Achieved	--		3.98 (3.26, 4.85)		2.75 (2.37, 3.19)	
10% Decrease	1.22 (1.19, 1.25)	<0.0001				
**Sex**						
Female (ref.)	1.00	0.0271	1.00	0.0202	1.00	0.0042
Male	0.84 (0.73, 0.98)		0.84 (0.72, 0.97)		0.81 (0.69, 0.93)	
**Age at MI (years)**	1.01 (1.01, 1.02)	<0.0001	1.01 (1.01, 1.02)	<0.0001	1.02 (1.01, 1.02)	<0.0001
**SIMD (NHS GGC)**						
1 [most]	1.38 (1.09, 1.75)	0.1206	1.36 (1.08, 1.72)	0.1425	1.28 (1.01, 1.61)	0.3288
2	1.23 (0.97, 1.56)		1.21 (0.96, 1.53)		1.17 (0.92, 1.47)	
3	1.27 (1.00, 1.62)		1.24 (0.98, 1.57)		1.22 (0.97, 1.55)	
4	1.24 (0.96, 1.59)		1.23 (0.96, 1.58)		1.20 (0.94, 1.54)	
5 [least] (ref.)	1.00		1.00		1.00	
**Year of MI**						
2009 (ref.)	1.00	0.9071	1.00	0.9764	1.00	0.7545
2010	1.11 (0.82, 1.51)		1.09 (0.80, 1.47)		1.19 (0.88, 1.61)	
2011	1.09 (0.82, 1.46)		1.06 (0.79, 1.41)		1.14 (0.86, 1.53)	
2012	1.17 (0.87, 1.56)		1.12 (0.84, 1.49)		1.24 (0.94, 1.66)	
2013	1.11 (0.83, 1.48)		1.05 (0.79, 1.40)		1.17 (0.88, 1.56)	
2014	1.19 (0.86, 1.64)		1.10 (0.80, 1.51)		1.24 (0.90, 1.70)	

For peer review only

Table s4: Models for LDL Target by Statin Adherence Thresholds (sensitivity inc. incomplete years of follow-up)

Model/Variable	Continuous Adherence		<50% Adherence		<80% Adherence	
	Odds Ratio (95% CI)	p-value	Odds Ratio (95% CI)	p-value	Odds Ratio (95% CI)	p-value
Unadjusted Model						
**Adherence Target**						
Achieved (ref.)	--		1.00	<0.0001	1.00	<0.0001
Not Achieved	--		1.86 (1.65, 2.09)		1.53 (1.39, 1.69)	
10% Decrease	1.09 (1.07, 1.10)	<0.0001				
Adjusted Model						
**Adherence Target**						
Achieved (ref.)	--		1.00	<0.0001	1.00	<0.0001
Not Achieved	--		1.90 (1.68, 2.14)		1.59 (1.44, 1.76)	
10% Decrease	1.09 (1.07, 1.11)	<0.0001				
**Sex**						
Female (ref.)	1.00	0.0003	1.00	0.0002	1.00	0.0001
Male	0.83 (0.74, 0.91)		0.82 (0.74, 0.91)		0.81 (0.73, 0.90)	
**Age at MI (years)**	1.00 (0.99, 1.00)	0.0590	1.00 (0.99, 1.00)	0.0619	1.00 (0.99, 1.00)	0.1200
**SIMD (NHS GGC)**						
1 [most]	0.82 (0.69, 0.97)	<0.0001	0.81 (0.68, 0.96)	<0.0001	0.80 (0.67, 0.94)	<0.0001
2	1.26 (1.07, 1.49)		1.26 (1.07, 1.48)		1.24 (1.05, 1.45)	
3	1.35 (1.14, 1.59)		1.34 (1.14, 1.58)		1.33 (1.13, 1.57)	
4	1.52 (1.28, 1.80)		1.51 (1.27, 1.79)		1.50 (1.27, 1.78)	
5 [least] (ref.)	1.00		1.00		1.00	
**Year of MI**						
2009 (ref.)	1.00	<0.0001	1.00	<0.0001	1.00	<0.0001
2010	1.11 (0.93, 1.33)		1.11 (0.93, 1.33)		1.12 (0.94, 1.34)	
2011	1.33 (1.12, 1.58)		1.32 (1.11, 1.57)		1.34 (1.13, 1.60)	
2012	1.33 (1.12, 1.58)		1.32 (1.10, 1.57)		1.35 (1.14, 1.61)	
2013	1.51 (1.27, 1.80)		1.49 (1.26, 1.78)		1.54 (1.29, 1.83)	
2014	3.90 (3.24, 4.71)		3.83 (3.18, 4.62)		3.93 (3.26, 4.73)	

For peer review only

Table s5: Models for All-Cause and CVD Mortality by Lipid Targets

	Non-HDL Target				LDL Target			
	All-Cause		Circulatory		All-Cause		Circulatory	
**Model/Variable**	**Hazard Ratio (95% CI)**	**p-value**	**Hazard Ratio (95% CI)**	**p-value**	**Hazard Ratio (95% CI)**	**p-value**	**Hazard Ratio (95% CI)**	**p-value**
Unadjusted Model								
**Lipid Target**								
Achieved (ref.)	1.00	<0.0001	1.00	<0.0001	1.00	<0.0001	1.00	0.0013
Not Achieved	2.16 (1.87, 2.49)		2.21 (1.72, 2.85)		1.27 (1.16, 1.39)		1.29 (1.11, 1.51)	
Adjusted Model								
**Lipid Target**								
Achieved (ref.)	1.00	<0.0001	1.00	<0.0001	1.00	<0.0001	1.00	0.0012
Not Achieved	1.76 (1.52, 2.03)		1.82 (1.41, 2.34)		1.32 (1.20, 1.44)		1.30 (1.11, 1.52)	
**Sex**								
Female (ref.)	1.00	0.0014	1.00	0.0101	1.00	0.0361	1.00	0.0062
Male	1.26 (1.09, 1.46)		1.38 (1.08, 1.78)		1.09 (1.01, 1.18)		1.22 (1.06, 1.40)	
**Age at MI (years)**	1.08 (1.08, 1.09)	<0.0001	1.08 (1.07, 1.09)	<0.0001	1.08 (1.08, 1.09)	<0.0001	1.08 (1.08, 1.09)	<0.0001
**SIMD (NHS GGC)**								
1 [most]	1.95 (1.53, 2.49)	<0.0001	1.66 (1.07, 2.58)	0.0286	1.92 (1.68, 2.20)	<0.0001	2.01 (1.58, 2.56)	<0.0001
2	1.56 (1.23, 1.99)		1.65 (1.08, 2.52)		1.66 (1.45, 1.90)		1.71 (1.34, 2.18)	
3	1.60 (1.26, 2.03)		1.91 (1.26, 2.90)		1.57 (1.37, 1.80)		1.75 (1.38, 2.23)	
4	1.22 (0.95, 1.57)		1.46 (0.95, 2.26)		1.32 (1.14, 1.52)		1.53 (1.19, 1.97)	
5 [least] (ref.)	1.00		1.00		1.00		1.00	
**Year of MI**								
2009 (ref.)	1.00	0.0441	1.00	0.4666	1.00	<0.0001	1.00	0.0570
2010	0.90 (0.70, 1.16)		0.90 (0.55, 1.46)		1.01 (0.90, 1.14)		1.04 (0.83, 1.30)	
2011	0.79 (0.61, 1.02)		1.14 (0.71, 1.83)		0.94 (0.83, 1.06)		1.20 (0.96, 1.50)	
2012	0.76 (0.58, 0.99)		1.22 (0.76, 1.98)		0.87 (0.76, 0.99)		1.33 (1.06, 1.68)	
2013	0.85 (0.64, 1.11)		1.35 (0.82, 2.23)		0.83 (0.72, 0.96)		1.31 (1.02, 1.68)	
2014	0.57 (0.39, 0.83)		1.00 (0.53, 1.90)		0.64 (0.52, 0.79)		0.98 (0.69, 1.39)	

For peer review only

Table s6: Models for Overall Mortality by Statin Adherence Thresholds

Model/Variable	Continuous Adherence		50% Adherence		80% Adherence	
	Hazard Ratio (95% CI)	p-value	Hazard Ratio (95% CI)	p-value	Hazard Ratio (95% CI)	p-value
Unadjusted Model						
**Adherence Target**						
Achieved (ref.)	--		1.00	<0.0001	1.00	<0.0001
Not Achieved	--		1.58 (1.44, 1.73)		1.53 (1.42, 1.66)	
10% Decrease	1.07 (1.06, 1.08)	<0.0001				
Adjusted Model						
**Adherence Target**						
Achieved (ref.)	--		1.00	<0.0001	1.00	<0.0001
Not Achieved	--		1.24 (1.13, 1.37)		1.37 (1.26, 1.48)	
10% Decrease	1.03 (1.02, 1.05)	<0.0001				
**Sex**						
Female (ref.)	1.00	0.0343	1.00	0.0448	1.00	0.0337
Male	1.09 (1.01, 1.18)		1.08 (1.00, 1.17)		1.09 (1.01, 1.18)	
**Age at MI (years)**	1.08 (1.08, 1.09)	<0.0001	1.08 (1.08, 1.09)	<0.0001	1.08 (1.08, 1.09)	<0.0001
**SIMD (NHS GGC)**						
1 [most]	1.91 (1.67, 2.18)	<0.0001	1.91 (1.67, 2.18)	<0.0001	1.90 (1.67, 2.17)	<0.0001
2	1.69 (1.49, 1.94)		1.69 (1.48, 1.93)		1.70 (1.49, 1.94)	
3	1.63 (1.43, 1.86)		1.63 (1.43, 1.86)		1.62 (1.42, 1.86)	
4	1.34 (1.16, 1.54)		1.34 (1.17, 1.54)		1.34 (1.16, 1.54)	
5 [least] (ref.)	1.00		1.00		1.00	
**Year of MI**						
2009 (ref.)	1.00	0.0031	1.00	0.0033	1.00	0.0078
2010	1.01 (0.90, 1.13)		1.01 (0.90, 1.13)		1.01 (0.90, 1.14)	
2011	0.93 (0.82, 1.04)		0.93 (0.82, 1.04)		0.93 (0.83, 1.05)	
2012	0.93 (0.82, 1.06)		0.93 (0.82, 1.06)		0.94 (0.83, 1.07)	
2013	0.83 (0.72, 0.96)		0.83 (0.72, 0.96)		0.85 (0.74, 0.98)	
2014	0.73 (0.60, 0.89)		0.73 (0.61, 0.89)		0.74 (0.61, 0.90)	

For peer review only

Table s7: Models for CVD Mortality by Statin Adherence Thresholds

Model/Variable	Continuous Adherence		50% Adherence		80% Adherence	
	Hazard Ratio (95% CI)	p-value	Hazard Ratio (95% CI)	p-value	Hazard Ratio (95% CI)	p-value
Unadjusted Model						
**Adherence Target**						
Achieved (ref.)	--		1.00	<0.0001	1.00	<0.0001
Not Achieved	--		1.60 (1.36, 1.88)		1.43 (1.25, 1.64)	
10% Decrease	1.06 (1.04, 1.09)	<0.0001				
Adjusted Model						
**Adherence Target**						
Achieved (ref.)	--		1.00	0.0116	1.00	0.0003
Not Achieved	--		1.24 (1.05, 1.46)		1.29 (1.12, 1.48)	
10% Decrease	1.03 (1.01, 1.05)	0.0072				
**Sex**						
Female (ref.)	1.00	0.0039	1.00	0.0042	1.00	0.0039
Male	1.23 (1.07, 1.41)		1.22 (1.07, 1.40)		1.23 (1.07, 1.41)	
**Age at MI (years)**	1.08 (1.08, 1.09)	<0.0001	1.09 (1.08, 1.09)	<0.0001	1.08 (1.08, 1.09)	<0.0001
**SIMD (NHS GGC)**						
1 [most]	1.70 (1.35, 2.14)	<0.0001	1.70 (1.36, 2.14)	<0.0001	1.70 (1.35, 2.14)	<0.0001
2	1.56 (1.25, 1.96)		1.56 (1.24, 1.95)		1.56 (1.25, 1.96)	
3	1.53 (1.22, 1.92)		1.53 (1.22, 1.92)		1.52 (1.21, 1.91)	
4	1.36 (1.08, 1.73)		1.37 (1.08, 1.73)		1.36 (1.08, 1.73)	
5 [least] (ref.)	1.00		1.00		1.00	
**Year of MI**						
2009 (ref.)	1.00	0.0005	1.00	0.0006	1.00	0.0004
2010	0.98 (0.79, 1.22)		0.98 (0.79, 1.22)		0.98 (0.79, 1.23)	
2011	1.23 (0.99, 1.53)		1.23 (0.99, 1.53)		1.24 (1.00, 1.54)	
2012	1.51 (1.21, 1.88)		1.50 (1.21, 1.88)		1.52 (1.22, 1.90)	
2013	1.22 (0.95, 1.56)		1.22 (0.95, 1.56)		1.24 (0.97, 1.59)	
2014	0.96 (0.68, 1.36)		0.96 (0.68, 1.36)		0.97 (0.69, 1.38)	

For peer review only

A – Overall Mortality

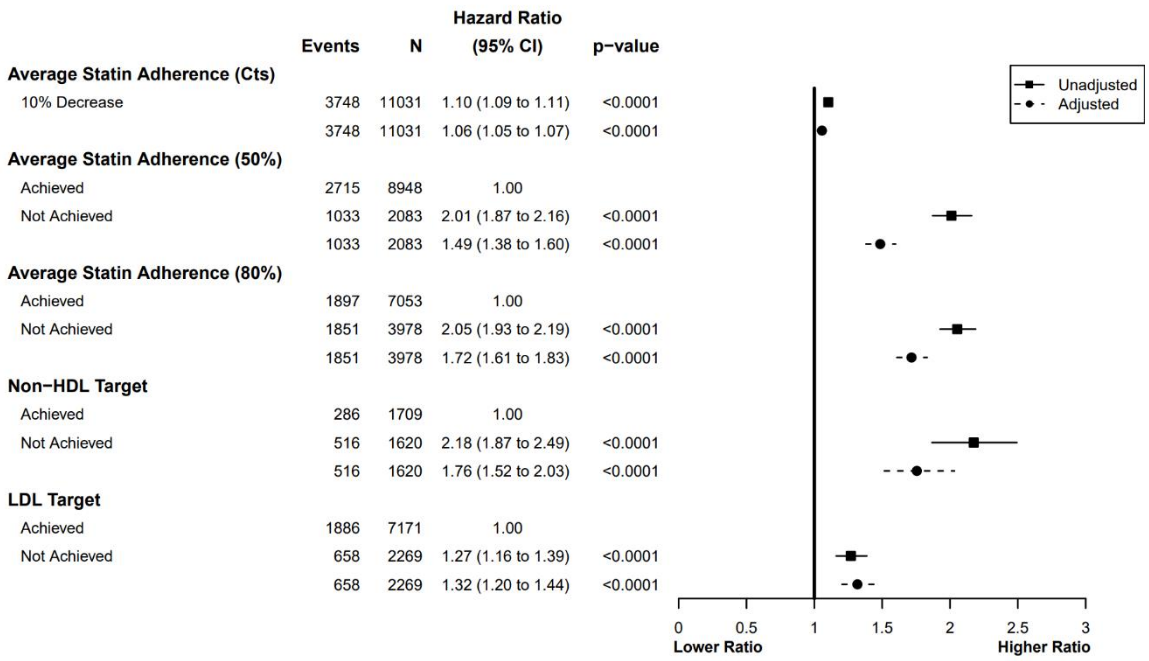

B – CVD Mortality

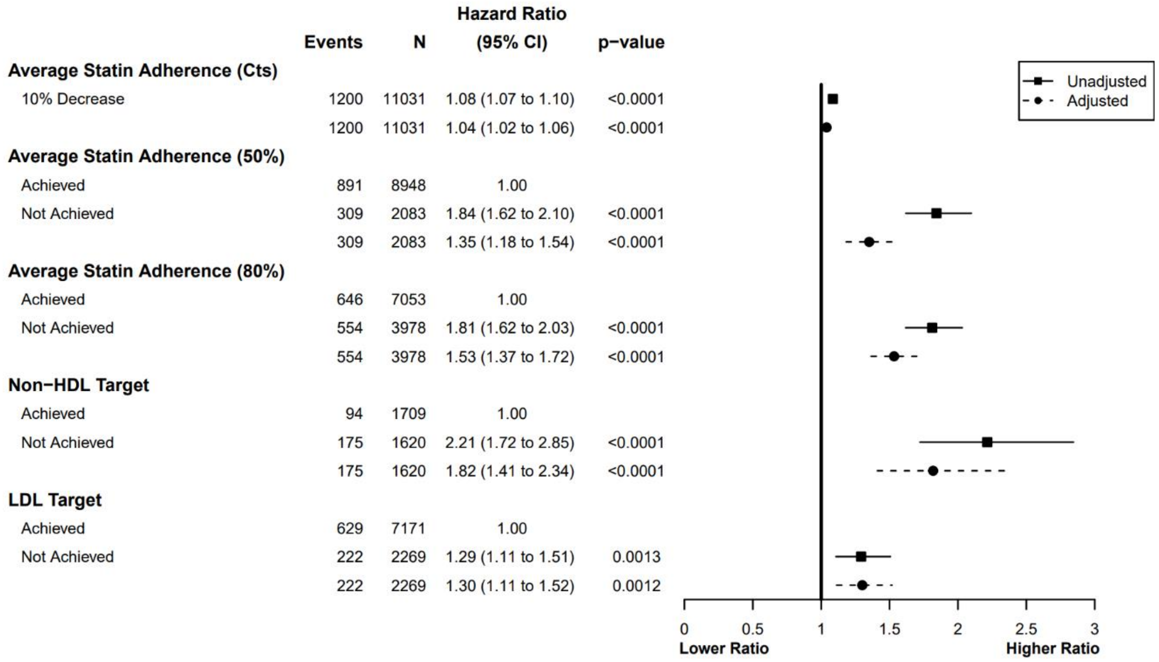

Figure s1: Hazard Ratios of A) All-Cause and B) CVD Mortality by Average Statin Adherence and Achievement of Plasma Lipid Targets (from NICE and ESC Recommendations) (Sensitivity inc. incomplete years of follow up)

Table s8: Models for Overall Mortality by Statin Adherence Thresholds (sensitivity inc. incomplete years of follow up)

	Continuous Adherence		50% Adherence		80% Adherence	
**Model/Variable**	**Hazard Ratio (95% CI)**	**p-value**	**Hazard Ratio (95% CI)**	**p-value**	**Hazard Ratio (95% CI)**	**p-value**
Unadjusted Model						
**Adherence Target**						
Achieved (ref.)	--		1.00	<0.0001	1.00	<0.0001
Not Achieved	--		2.01 (1.87, 2.16)		2.05 (1.93, 2.19)	
10% Decrease	1.10 (1.09, 1.11)	<0.0001				
Adjusted Model						
**Adherence Target**						
Achieved (ref.)	--		1.00	<0.0001	1.00	<0.0001
Not Achieved	--		1.49 (1.38, 1.60)		1.72 (1.61, 1.83)	
10% Decrease	1.06 (1.05, 1.07)	<0.0001				
**Sex**						
Female (ref.)	1.00	0.0037	1.00	0.0052	1.00	0.0027
Male	1.10 (1.03, 1.18)		1.10 (1.03, 1.18)		1.11 (1.04, 1.19)	
**Age at MI (years)**	1.08 (1.08, 1.08)	<0.0001	1.08 (1.08, 1.08)	<0.0001	1.08 (1.08, 1.08)	<0.0001
**SIMD (NHS GGC)**						
1 [most]	1.76 (1.58, 1.97)	<0.0001	1.77 (1.57, 1.96)	<0.0001	1.75 (1.56, 1.95)	<0.0001
2	1.56 (1.39, 1.74)		1.55 (1.39, 1.74)		1.56 (1.39, 1.74)	
3	1.48 (1.32, 1.65)		1.47 (1.31, 1.65)		1.47 (1.31, 1.65)	
4	1.29 (1.15, 1.45)		1.29 (1.15, 1.45)		1.28 (1.14, 1.44)	
5 [least] (ref.)	1.00		1.00		1.00	
**Year of MI**						
2009 (ref.)	1.00	0.0013	1.00	0.0012	1.00	0.0057
2010	0.95 (0.86, 1.05)		0.95 (0.86, 1.06)		0.97 (0.87, 1.07)	
2011	0.94 (0.85, 1.04)		0.94 (0.85, 1.04)		0.95 (0.86, 1.06)	
2012	0.93 (0.83, 1.03)		0.93 (0.83, 1.03)		0.95 (0.85, 1.05)	
2013	0.85 (0.75, 0.95)		0.85 (0.75, 0.95)		0.87 (0.78, 0.98)	
2014	0.74 (0.64, 0.86)		0.74 (0.64, 0.86)		0.76 (0.65, 0.88)	

For peer review only

Table s9: Models for CVD Mortality by Statin Adherence Thresholds (sensitivity inc. incomplete years of follow up)

	Continuous Adherence		50% Adherence		80% Adherence	
**Model/Variable**	**Hazard Ratio (95% CI)**	**p-value**	**Hazard Ratio (95% CI)**	**p-value**	**Hazard Ratio (95% CI)**	**p-value**
Unadjusted Model						
**Adherence Target**						
Achieved (ref.)	--		1.00	<0.0001	1.00	<0.0001
Not Achieved	--		1.84 (1.62, 2.10)		1.81 (1.62, 2.03)	
10% Decrease	1.09 (1.07, 1.10)	<0.0001				
Adjusted Model						
**Adherence Target**						
Achieved (ref.)	--		1.00	0.0001	1.00	<0.0001
Not Achieved	--		1.35 (1.18, 1.54)		1.53 (1.37, 1.72)	
10% Decrease	1.04 (1.02, 1.06)	<0.0001				
**Sex**						
Female (ref.)	1.00	0.0002	1.00	0.0002	1.00	0.0001
Male	1.26 (1.11, 1.42)		1.25 (1.11, 1.41)		1.26 (1.12, 1.42)	
**Age at MI (years)**	1.08 (1.08, 1.09)	<0.0001	1.08 (1.08, 1.09)	<0.0001	1.08 (1.08, 1.09)	<0.0001
**SIMD (NHS GGC)**						
1 [most]	1.77 (1.45, 2.16)	<0.0001	1.77 (1.45, 2.16)	<0.0001	1.76 (1.45, 2.15)	<0.0001
2	1.62 (1.33, 1.97)		1.62 (1.33, 1.97)		1.63 (1.34, 1.98)	
3	1.51 (1.24, 1.85)		1.51 (1.24, 1.84)		1.51 (1.24, 1.84)	
4	1.34 (1.09, 1.65)		1.34 (1.09, 1.65)		1.33 (1.08, 1.64)	
5 [least] (ref.)	1.00		1.00		1.00	
**Year of MI**						
2009 (ref.)	1.00	0.0005	1.00	<0.0001	1.00	<0.0001
2010	0.95 (0.78, 1.16)		0.95 (0.78, 1.16)		0.96 (0.79, 1.17)	
2011	1.15 (0.95, 1.39)		1.15 (0.95, 1.39)		1.16 (0.96, 1.41)	
2012	1.47 (1.21, 1.78)		1.47 (1.21, 1.78)		1.49 (1.23, 1.81)	
2013	1.35 (1.10, 1.66)		1.35 (1.10, 1.66)		1.38 (1.12, 1.70)	
2014	1.20 (0.92, 1.56)		1.20 (0.92, 1.56)		1.22 (0.94, 1.58)	

For peer review only

Table s10: Models for MIs by Lipid Targets

Model/Variable	Non-HDL Target: Odds Ratio (95% CI)	Non-HDL Target: p-value	LDL Target: Odds Ratio (95% CI)	LDL Target: p-value
Unadjusted Model				
**Lipid Target**				
Achieved (ref.)	1.00	0.0127	1.00	0.8776
Not Achieved	1.21 (1.04, 1.40)		1.01 (0.91, 1.12)	
Adjusted Model				
**Lipid Target**				
Achieved (ref.)	1.00	0.0161	1.00	0.9419
Not Achieved	1.20 (1.03, 1.40)		1.00 (0.90, 1.12)	
**Sex**				
Female (ref.)	1.00	0.6005	1.00	0.9146
Male	1.04 (0.89, 1.22)		1.01 (0.91, 1.10)	
**Age at MI (years)**	1.00 (1.00, 1.01)	0.2742	1.01 (1.00, 1.01)	0.0007
**SIMD (NHS GGC)**				
1 [most]	0.89 (0.70, 1.14)	0.0763	1.21 (1.04, 1.40)	0.0200
2	0.99 (0.79, 1.26)		1.08 (0.93, 1.25)	
3	0.97 (0.76, 1.24)		1.15 (0.99, 1.33)	
4	0.72 (0.56, 0.95)		0.98 (0.84, 1.15)	
5 [least] (ref.)	1.00		1.00	
**Year of MI**				
2009 (ref.)	1.00	0.4627	1.00	0.0006
2010	0.99 (0.72, 1.36)		1.00 (0.86, 1.16)	
2011	0.98 (0.72, 1.34)		1.08 (0.93, 1.26)	
2012	1.16 (0.86, 1.56)		1.30 (1.12, 1.51)	
2013	1.19 (0.88, 1.61)		1.25 (1.07, 1.45)	
2014	1.06 (0.76, 1.49)		1.14 (0.95, 1.37)	

For peer review only

Table s11: Models for MIs by Statin Adherence Thresholds

Model/Variable	Continuous Adherence		50% Adherence		80% Adherence	
	Odds Ratio (95% CI)	p-value	Odds Ratio (95% CI)	p-value	Odds Ratio (95% CI)	p-value
Unadjusted Model						
**Adherence Target**						
Achieved (ref.)	--		1.00	0.1221	1.00	0.1309
Not Achieved	--		0.91 (0.81, 1.03)		1.07 (0.98, 1.18)	
10% Decrease	1.00 (0.98, 1.01)	0.5141				
Adjusted Model						
**Adherence Target**						
Achieved (ref.)	--		1.00	0.0803	1.00	0.1346
Not Achieved	--		0.90 (0.79, 1.01)		1.07 (0.98, 1.18)	
10% Decrease	0.99 (0.98, 1.01)	0.4278				
**Sex**						
Female (ref.)	1.00	0.7230	1.00	0.7595	1.00	0.6382
Male	1.02 (0.93, 1.12)		1.01 (0.92, 1.11)		1.02 (0.93, 1.12)	
**Age at MI (years)**	1.00 (1.00, 1.01)	0.0388	1.00 (1.00, 1.01)	0.0337	1.00 (1.00, 1.01)	0.0462
**SIMD (NHS GGC)**						
1 [most]	1.16 (1.01, 1.34)	0.0380	1.16 (1.01, 1.34)	0.0404	1.16 (1.01, 1.34)	0.0385
2	1.07 (0.92, 1.23)		1.07 (0.92, 1.23)		1.07 (0.93, 1.24)	
3	1.12 (0.96, 1.29)		1.11 (0.96, 1.29)		1.11 (0.96, 1.29)	
4	0.95 (0.81, 1.11)		0.95 (0.81, 1.11)		0.95 (0.81, 1.11)	
5 [least] (ref.)	1.00		1.00		1.00	
**Year of MI**						
2009 (ref.)	1.00	0.0003	1.00	0.0002	1.00	0.0003
2010	0.98 (0.84, 1.14)		0.98 (0.84, 1.14)		0.98 (0.84, 1.14)	
2011	1.06 (0.91, 1.24)		1.06 (0.91, 1.24)		1.06 (0.91, 1.24)	
2012	1.30 (1.12, 1.51)		1.30 (1.12, 1.51)		1.30 (1.12, 1.50)	
2013	1.22 (1.05, 1.42)		1.23 (1.06, 1.43)		1.23 (1.06, 1.43)	
2014	1.08 (0.90, 1.28)		1.08 (0.91, 1.28)		1.08 (0.91, 1.29)	

For peer review only

	Events	N	Odds Ratio (95% CI)	p−value
**Average Statin Adherence (Cts)**				
10% Decrease	3265	11031	0.99 (0.98 to 1.01)	0.3392
	3265	11031	0.99 (0.98 to 1.00)	0.2279
**Average Statin Adherence (50%)**				
Achieved	2684	8948	1.00	
Not Achieved	581	2083	0.90 (0.81 to 1.00)	0.0572
	581	2083	0.89 (0.80 to 0.99)	0.0255
**Average Statin Adherence (80%)**				
Achieved	2049	7053	1.00	
Not Achieved	1216	3978	1.08 (0.99 to 1.17)	0.1305
	1216	3978	1.07 (0.98 to 1.16)	0.1305
**Non−HDL Target**				
Achieved	490	1709	1.00	
Not Achieved	529	1620	1.21 (1.04 to 1.40)	0.0127
	529	1620	1.20 (1.03 to 1.40)	0.0161
**LDL Target**				
Achieved	2159	7171	1.00	
Not Achieved	687	2269	1.01 (0.91 to 1.12)	0.8776
	687	2269	1.00 (0.90 to 1.12)	0.9419

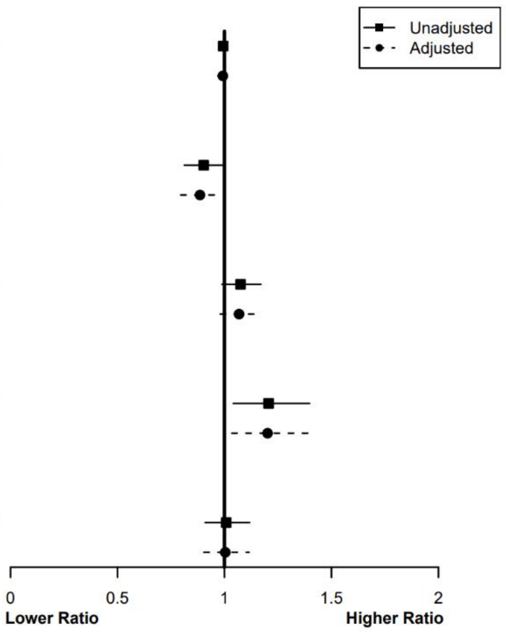

Figure s2: Odds of MIs During Follow Up by Average Statin Adherence and Achievement of Plasma Lipid Targets (from NICE and ESC Recommendations) (Sensitivity inc. incomplete years of follow up)

For peer review only

Table s12: Models for MIs by Average Statin Adherence Thresholds (sensitivity inc. incomplete years)

	Continuous Adherence		50% Adherence		80% Adherence	
**Model/Variable**	**Odds Ratio (95% CI)**	**p-value**	**Odds Ratio (95% CI)**	**p-value**	**Odds Ratio (95% CI)**	**p-value**
Unadjusted Model						
**Adherence Target**						
Achieved (ref.)	--		1.00	0.0572	1.00	0.0943
Not Achieved	--		0.90 (0.81, 1.00)		1.08 (0.99, 1.17)	
10% Decrease	0.99 (0.98, 1.01)	0.3392				
Adjusted Model						
**Adherence Target**						
Achieved (ref.)	--		1.00	0.0255	1.00	0.1305
Not Achieved	--		0.89 (0.80, 0.99)		1.07 (0.98, 1.16)	
10% Decrease	1.00 (0.98, 1.00)	0.2279				
**Sex**						
Female (ref.)	1.00	0.9381	1.00	0.9824	1.00	0.8353
Male	1.00 (0.92, 1.09)		1.00 (0.92, 1.09)		1.01 (0.93, 1.10)	
**Age at MI (years)**	1.00 (1.00, 1.01)	0.0310	1.00 (1.00, 1.01)	0.0226	1.00 (1.00, 1.01)	0.0553
**SIMD (NHS GGC)**						
1 [most]	1.16 (1.01, 1.33)	0.0304	1.16 (1.01, 1.33)	0.0315	1.16 (1.01, 1.33)	0.0307
2	1.09 (0.95, 1.25)		1.09 (0.95, 1.25)		1.09 (0.95, 1.25)	
3	1.11 (0.96, 1.27)		1.11 (0.97, 1.27)		1.11 (0.96, 1.27)	
4	0.95 (0.82, 1.10)		0.95 (0.82, 1.10)		0.95 (0.82, 1.10)	
5 [least] (ref.)	1.00		1.00		1.00	
**Year of MI**						
2009 (ref.)	1.00	0.0024	1.00	0.0020	1.00	0.0025
2010	0.99 (0.85, 1.14)		0.99 (0.85, 1.14)		0.99 (0.86, 1.14)	
2011	1.06 (0.92, 1.22)		1.06 (0.92, 1.22)		1.06 (0.92, 1.22)	
2012	1.24 (1.08, 1.42)		1.24 (1.08, 1.43)		1.24 (1.08, 1.42)	
2013	1.20 (1.05, 1.39)		1.21 (1.05, 1.39)		1.21 (1.05, 1.39)	
2014	1.08 (0.91, 1.27)		1.08 (0.92, 1.28)		1.08 (0.92, 1.28)	

For peer review only

STROBE Statement—checklist of items that should be included in reports of observational studies

	Item No	Recommendation	Page No
**Title and abstract**	1	(*a*) Indicate the study's design with a commonly used term in the title or the abstract	**1, 2**
		(*b*) Provide in the abstract an informative and balanced summary of what was done and what was found	**2**
**Introduction**			
Background/rationale	2	Explain the scientific background and rationale for the investigation being reported	**4**
Objectives	3	State specific objectives, including any prespecified hypotheses	**4**
**Methods**			
Study design	4	Present key elements of study design early in the paper	**5**
Setting	5	Describe the setting, locations, and relevant dates, including periods of recruitment, exposure, follow-up, and data collection	**5**
Participants	6	(*a*) *Cohort study*—Give the eligibility criteria, and the sources and methods of selection of participants. Describe methods of follow-up<br>*Case-control study*—Give the eligibility criteria, and the sources and methods of case ascertainment and control selection. Give the rationale for the choice of cases and controls<br>*Cross-sectional study*—Give the eligibility criteria, and the sources and methods of selection of participants	**5, Fig 1**
		(*b*) *Cohort study*—For matched studies, give matching criteria and number of exposed and unexposed<br>*Case-control study*—For matched studies, give matching criteria and the number of controls per case	**N/A**
Variables	7	Clearly define all outcomes, exposures, predictors, potential confounders, and effect modifiers. Give diagnostic criteria, if applicable	**5-7**
Data sources/ measurement	8*	For each variable of interest, give sources of data and details of methods of assessment (measurement). Describe comparability of assessment methods if there is more than one group	**5-7**
Bias	9	Describe any efforts to address potential sources of bias	**7**
Study size	10	Explain how the study size was arrived at	**5, Fig 1**
Quantitative variables	11	Explain how quantitative variables were handled in the analyses. If applicable, describe which groupings were chosen and why	**5-7**
Statistical methods	12	(*a*) Describe all statistical methods, including those used to control for confounding	**7**
		(*b*) Describe any methods used to examine subgroups and interactions	**7**
		(*c*) Explain how missing data were addressed	**7**
		(*d*) *Cohort study*—If applicable, explain how loss to follow-up was addressed<br>*Case-control study*—If applicable, explain how matching of cases and controls was addressed	**N/A**

For peer review only - http://bmjopen.bmj.com/site/about/guidelines.xhtml

		Cross-sectional study—If applicable, describe analytical methods taking account of sampling strategy	
		(*e*) Describe any sensitivity analyses	**7**
**Results**			
Participants	13*	(a) Report numbers of individuals at each stage of study—eg numbers potentially eligible, examined for eligibility, confirmed eligible, included in the study, completing follow-up, and analysed	**Fig 1**
		(b) Give reasons for non-participation at each stage	**Fig 1**
		(c) Consider use of a flow diagram	**Fig 1**
Descriptive data	14*	(a) Give characteristics of study participants (eg demographic, clinical, social) and information on exposures and potential confounders	**7,8, Tab 1**
		(b) Indicate number of participants with missing data for each variable of interest	**Tab 1**
		(c) *Cohort study*—Summarise follow-up time (eg, average and total amount)	**7**
Outcome data	15*	*Cohort study*—Report numbers of outcome events or summary measures over time	**Fig 2 Fig 3**
		Case-control study—Report numbers in each exposure category, or summary measures of exposure	**N/A**
		Cross-sectional study—Report numbers of outcome events or summary measures	**N/A**
Main results	16	(*a*) Give unadjusted estimates and, if applicable, confounder-adjusted estimates and their precision (eg, 95% confidence interval). Make clear which confounders were adjusted for and why they were included	**8, 9 Fig 2, Fig 3**
		(*b*) Report category boundaries when continuous variables were categorized	**6,7**
		(*c*) If relevant, consider translating estimates of relative risk into absolute risk for a meaningful time period	**N/A**
Other analyses	17	Report other analyses done—eg analyses of subgroups and interactions, and sensitivity analyses	**8, 9, Supp**
**Discussion**			
Key results	18	Summarise key results with reference to study objectives	**9**
Limitations	19	Discuss limitations of the study, taking into account sources of potential bias or imprecision. Discuss both direction and magnitude of any potential bias	**10-11**
Interpretation	20	Give a cautious overall interpretation of results considering objectives, limitations, multiplicity of analyses, results from similar studies, and other relevant evidence	**10-12**
Generalisability	21	Discuss the generalisability (external validity) of the study results	**11**
**Other information**			
Funding	22	Give the source of funding and the role of the funders for the present study and, if applicable, for the original study on which the present article is based	**12**

*Give information separately for cases and controls in case-control studies and, if applicable, for exposed and unexposed groups in cohort and cross-sectional studies.

Note: An Explanation and Elaboration article discusses each checklist item and gives methodological background and published examples of transparent reporting. The STROBE checklist is best used in conjunction with this article (freely available on the Web sites of PLoS Medicine at http://www.plosmedicine.org/, Annals of Internal Medicine at

For peer review only - http://bmjopen.bmj.com/site/about/guidelines.xhtml

http://www.annals.org/, and Epidemiology at http://www.epidem.com/). Information on the STROBE Initiative is available at www.strobe-statement.org.

For peer review only

For peer review only - http://bmjopen.bmj.com/site/about/guidelines.xhtml

Associations of statin adherence and lipid targets with adverse outcomes in myocardial infarction survivors: a retrospective cohort study

Journal:	*BMJ Open*
Manuscript ID	bmjopen-2021-054893.R1
Article Type:	Original research
Date Submitted by the Author:	02-Sep-2021
Complete List of Authors:	Brown, Rosemary; University of Glasgow, Institute of Cardiovascular and Medical Sciences<br>Lewsey, Jim; University of Glasgow<br>Wild, Sarah; The University of Edinburgh, Public Health Sciences<br>Logue, Jennifer; Lancaster University, Lancaster Medical School<br>Welsh, Paul; University of Glasgow, Institute of Cardiovascular and Medical Sciences; University Of Glasgow
**Primary Subject Heading**:	Cardiovascular medicine
Secondary Subject Heading:	Epidemiology
Keywords:	Cardiac Epidemiology < CARDIOLOGY, Myocardial infarction < CARDIOLOGY, EPIDEMIOLOGY

SCHOLARONE™
Manuscripts

I, the Submitting Author has the right to grant and does grant on behalf of all authors of the Work (as defined in the below author licence), an exclusive licence and/or a non-exclusive licence for contributions from authors who are: i) UK Crown employees; ii) where BMJ has agreed a CC-BY licence shall apply, and/or iii) in accordance with the terms applicable for US Federal Government officers or employees acting as part of their official duties; on a worldwide, perpetual, irrevocable, royalty-free basis to BMJ Publishing Group Ltd ("BMJ") its licensees and where the relevant Journal is co-owned by BMJ to the co-owners of the Journal, to publish the Work in this journal and any other BMJ products and to exploit all rights, as set out in our licence.

The Submitting Author accepts and understands that any supply made under these terms is made by BMJ to the Submitting Author unless you are acting as an employee on behalf of your employer or a postgraduate student of an affiliated institution which is paying any applicable article publishing charge ("APC") for Open Access articles. Where the Submitting Author wishes to make the Work available on an Open Access basis (and intends to pay the relevant APC), the terms of reuse of such Open Access shall be governed by a Creative Commons licence – details of these licences and which Creative Commons licence will apply to this Work are set out in our licence referred to above.

Other than as permitted in any relevant BMJ Author's Self Archiving Policies, I confirm this Work has not been accepted for publication elsewhere, is not being considered for publication elsewhere and does not duplicate material already published. I confirm all authors consent to publication of this Work and authorise the granting of this licence.

For peer review only

Associations of statin adherence and lipid targets with adverse outcomes in myocardial infarction survivors: a retrospective cohort study

Authors:

Rosemary Brown[a], Jim Lewsey[b], Sarah Wild[c], Jennifer Logue *[d], Paul Welsh*[a]

*Joint senior authors

[a]BHF Glasgow Cardiovascular Research Centre, Institute of Cardiovascular and Medical Science, University of Glasgow, Glasgow, UK.

[b]Institute of Health & Wellbeing, University of Glasgow, Glasgow, UK.

[c]Usher Institute, University of Edinburgh, Edinburgh, UK.

[d]Lancaster Medical School, Health Innovation One, Sir John Fisher Drive, Lancaster University, Lancaster, UK.

Corresponding Author:

Paul Welsh

Institute of Cardiovascular and Medical Sciences, University of Glasgow, BHF Glasgow Cardiovascular Research Centre, 126 University Place, Glasgow G12 8TA, UK.

Tel: +44 (0)141 330 1722

Email address: paul.welsh@glasgow.ac.uk

Word Count (Main Body): 3,709

Number of Tables and Figures: 4

I, the Submitting Author has the right to grant and does grant on behalf of all authors of the Work (as defined in the below author licence), an exclusive licence and/or a non-exclusive licence for contributions from authors who are: i) UK Crown employees; ii) where BMJ has agreed a CC-BY licence shall apply, and/or iii) in accordance with the terms applicable for US Federal Government officers or employees acting as part of their official duties; on a worldwide, perpetual, irrevocable, royalty-free basis to BMJ Publishing Group Ltd ("BMJ") its licensees and where the relevant Journal is co-owned by BMJ to the co-owners of the Journal, to publish the Work in BMJ Open and any other BMJ products and to exploit all rights, as set out in our licence.

For peer review only - http://bmjopen.bmj.com/site/about/guidelines.xhtml

For peer review only

Abstract

Objectives To examine associations between statin adherence and lipid target achievement in myocardial infarction (MI) survivors, and their associations with mortality and recurrent MIs.

Design Retrospective cohort study using linked clinical records within the National Health Service Greater Glasgow and Clyde (NHS GGC) Data Safe Haven.

Setting Routine clinical practice in the NHS GGC area between January 2009 and July 2017.

Participants Patients ≥18 years who experienced a non-fatal MI hospital admission (ICD10: I21, I22) between January 2009 and July 2014 (n=11,031), followed up from the date of MI admission until July 2017 or death, whichever occurred first.

Primary and Secondary Outcome Measures Statin adherence was estimated using encashed prescriptions, and lipid results from routine biochemistry data. Primary lipid and statin adherence targets were LDL≤1.8mmol/L and adherence ≥50%, and were related to all-cause death, deaths due to cardiovascular disease (CVD) (ICD10: I00-I99 as the underlying cause), and recurrent MI in unadjusted models and models adjusting for age, sex, socioeconomic deprivation, and year of MI.

Results Over 4.5 years follow-up, 76% achieved LDL≤1.8mmol/L, and 84.5% had average adherence ≥50%. Patients with adherence <50% had an increased risk of not meeting LDL≤1.8mmol/L, in adjusted models (OR (95% CI): 2.03 (1.78, 2.31), p<0.0001). In univariable models, not meeting LDL≤1.8mmol/L was associated with increased risks of all-cause mortality (HR 1.27 (1.16, 1.39), p<0.0001) and CVD mortality (1.29 (1.11, 1.51), p=0.0013). Adherence <50% was associated with increased risks of all-cause mortality (1.58 (1.44, 1.74), p<0.0001) and CVD mortality (1.60 (1.36, 1.88), p<0.0001). Adjustment for confounders did not abrogate these associations. Neither exposure was associated with recurrent MIs.

Conclusions Non-achievement of lipid and adherence targets are associated with increased risks of all-cause and CVD mortality. Further work is required to optimise their use to improve outcomes in clinical practice.

Abstract Word Count: 294/300

For peer review only - http://bmjopen.bmj.com/site/about/guidelines.xhtml

Strengths and limitations of this study

- Data were derived from routine clinical practice records within a large health board in the West of Scotland, with average duration of follow up of 4.5 years.
- The use of encashed prescriptions in estimating adherence may be stronger evidence of medicines use over issued prescriptions.
- In mortality analysis, time-window bias could have arisen as patients with events had reduced opportunities to achieve lipid targets or improve average statin adherence.
- Suitable baseline lipid results were substantially missing within secondary prevention populations (~70%), limiting the utility of percentage change targets in this analysis.

For peer review only

For peer review only - http://bmjopen.bmj.com/site/about/guidelines.xhtml

Introduction

Those with established CVD (i.e., secondary prevention) are universally identified and targeted in risk management strategies due to their markedly higher rates of further cardiovascular events and overall mortality [1,2]. A cornerstone of these strategies is lipid-lowering medications, where statins are the first-line therapy.

Adherence to these medications and attaining lipid targets are key components of a patient review to address their cardiovascular risk in Scotland and the rest of the UK [3,4]. Indeed, achievement of target lipid levels (set by clinical guidelines) has been associated with a reduced likelihood of further events and mortality [1,5]. The association between adherence and the risk of cardiovascular events has also been shown in primary and secondary prevention in observational data, although adherence was measured using prescriptions issued rather than by drugs dispensed [6,7].

There is evidence to suggest adherence is higher in secondary prevention than primary prevention [8], and analyses have found reasonable adherence in secondary prevention cohorts; between 68% and 75% had statin adherence ≥80% in the year following an event [9,10]. However, this was not translated into target lipid levels, where fewer than half of the patients achieved these in the same period [10]. Despite this, many of the risk factors associated with CVD have also been shown to be associated with adherence and achievement of target lipid levels. For example, men [11,12], and, in primary prevention, those with a diagnosis of diabetes and hypertension [12] are more likely to be adherent. In secondary prevention, however, insulin use has been associated with non-adherence [13], although in a separate secondary prevention cohort, those with a diabetes diagnosis were more likely to meet lipid targets [10]. Therefore, there superficially appears to be differences between lipid-lowering medication adherence and lipid target achievement. This leads to uncertainties in clinical guidelines and in clinical practice as to whether adherence or lipid targets are more useful surrogates of risk reduction [14].

This analysis, using routinely collected data available for a secondary prevention population representative of a UK health board region, therefore seeks to compare and contrast statin adherence and lipid target achievement, and compare their associations with mortality and recurrent MIs. This analysis also investigates which is more strongly associated with outcomes, and therefore relevant for clinical decision making.

For peer review only - http://bmjopen.bmj.com/site/about/guidelines.xhtml

Methods

Data

The data used for this analysis was a subset of an extract of all individuals in NHS Greater Glasgow (GGC) and Clyde, a UK National Health Service region in the West of Scotland, who had a lipid profile result or a prescription for a statin, ezetimibe, or PCSK9 inhibitor before 29th December 2017. This initial data extraction was performed by staff in the NHS GGC Data Safe Haven and contained data for 652,441 individuals (Project Reference: GSH/17/CA/012 PMCVD; NHS GGC Safe Haven approval covers governance and ethical approvals). Data extracted included demographics, laboratory results, dispensed prescriptions, hospital admissions, death certificates, and diabetes diagnoses.

To maximise completeness, patients were followed up between 1st January 2009 and 31st July 2017 (inclusive). We excluded patients who resided outside the region, as these individuals are likely to have travelled into the region for only some aspects of their care, and therefore a complete representation of their health status was unlikely. Patients who were <18 years, died before 1st January 2009, had multiple death certificates, or received apparent posthumous lipid tests or prescriptions for lipid-lowering medications more than six months posthumously were also excluded. Figure 1 provides an overview of the cohort derivation.

Patient and Public Involvement

Neither patients nor the public were involved in the design, conduct, reporting, or dissemination of this research.

Post MI Population

MIs were identified in linked hospital admission records (ICD10: I21, I22 in any diagnosis field) to identify patients who experienced an MI between 1st January 2009 and 31st July 2014. These patients were followed up from their first non-fatal MI admission until 31st July 2017 or death, whichever occurred first. Patients who only experienced fatal MIs (death occurred during admission or within 30 days of discharge), whose statin adherence was >200%, or were missing a socioeconomic deprivation index were removed. Year-long time windows were constructed for each patient, starting from their baseline admission date.

Statin Adherence

Statin prescription dispensing records were used to estimate patients' adherence. Dispensed dates were used as this was the first date that the patient could have possession of the medication.

For peer review only - http://bmjopen.bmj.com/site/about/guidelines.xhtml

Prescription end dates were calculated as the dispensed date plus the day-coverage dispensed, and quantities were adjusted to reflect dose availability where overlaps across time windows or death occurred. The Medication Possession Ratio (MPR) [15] was calculated as (number of doses dispensed in time window/length of time window)*100 for each full-year time window, and mean-averaged. Average adherence was considered continuously and dichotomously, using cut-offs at 50% [16] and 80% [15,17]. Given that alternate day dosing is often implemented instances of poor tolerance of medication [16], the 50% threshold was considered the primary exposure of interest.

Lipid Targets

Lipid profiles were collected as part of routine clinical practice in NHS Biochemistry laboratories. A lipid profile result consisted of five components: Total Cholesterol, Total Cholesterol:HDL Cholesterol, HDL Cholesterol, LDL Cholesterol, and Triglycerides. If a patient had multiple tests within a time window, the mean value of the tests was calculated. Non-HDL cholesterol was calculated as total cholesterol minus HDL cholesterol.

The primary lipid exposure of interest was the 2016 European Society of Cardiology (ESC) target for LDL cholesterol ≤1.8mmol/l [18], and the 2014 National Institute for Health and Care Excellence (NICE) target for a ≥40% reduction in non-HDL cholesterol [3] was a secondary lipid exposure of interest. The latter, though not formally recommended as a target, is also referred to within current Scottish guidelines, where achievement is considered indicative of adequate adherence to statin therapy [4]. To assess the attainment of the non-HDL target, a pre-MI statin-naïve baseline was required and was identified as the last lipid profile test a patient had before their baseline admission that was more than six months (182 days) after the end date of any previous statin prescription. The need for a baseline result limited the coverage of this exposure to 30%.

Outcomes

Underlying cause and date of death were obtained from linked death certificates and hospital discharge notices, with CVD deaths defined as ICD10: I00-I99. Patients missing a cause of death were assumed to have a non-CVD underlying cause of death. All MI events from linked hospital admission records (Scottish Morbidity Record (SMR01)) and death certificates before the end of follow-up were included as a separate outcome (ICD-10: I21, I22).

Other Covariates

For peer review only - http://bmjopen.bmj.com/site/about/guidelines.xhtml

For peer review only

Date of birth and sex was extracted from clinical records. For socioeconomic deprivation, the Scottish Index of Multiple Deprivation (SIMD) was obtained from the patients' residential postcode at baseline. Due to the higher number of the most deprived zones in this region than in Scotland in general [19], NHS GGC specific SIMD quintiles were derived to effectively assess the impact of deprivation within the post MI population of NHS GGC. The patient's age at MI was calculated using their date of birth and date of baseline MI. Prior MIs were defined as MI admissions occurring in the ten years before their baseline. The date of diagnosis and the type of diabetes was obtained from routine electronic diabetes records. The date of diagnosis was taken as the patient's first valid (i.e. after the patent's date of birth) date of entry in the diabetes register. Patients were considered to have had a diagnosis of diabetes at the time of their MI if this date was before their baseline MI admission. The type of diabetes was extracted, and if multiple types were listed during a patient's history, the most common diagnosis was taken.

Statistical Analysis

All analyses were performed using R v3.5.0 [20], and the 'forestplot' [21], 'survival' [22], and 'survminer' [23] packages. Continuous variables were summarised using mean and standard deviation and categorical variables with the number of observations (N) and percentages. As a patient's average adherence was calculated using complete years of follow-up, only patients who survived ≥1 year were included in analyses including adherence. For analyses including lipid targets, patients were included if they reported ≥1 test result during follow-up and were classified as meeting a target if they achieved it at any stage during follow-up.

Due to the potential for multiple recurrent MIs to occur in an individual, and the relatively short one-year time windows modelled, logistic regression was used to model the associations between average adherence and the achievement of lipid targets, and the association between adherence, and lipid targets, with recurrent MIs. For mortality, Cox regression was conducted, and the proportional hazards assumption was checked by visual inspection of the partial Schoenfeld residuals. For all outcomes, unadjusted and adjusted (for age at MI, sex, deprivation quintile, and year of MI) models were generated, using available case analysis for each exposure. Where adherence was considered dichotomously, and for the lipid targets, achievement of the threshold or target was the reference. A sensitivity analysis for adherence was also conducted by including events that occurred in the first year of follow-up. For all analyses, a p-value <0.05 was considered statistically significant.

For peer review only - http://bmjopen.bmj.com/site/about/guidelines.xhtml

Results

Baseline Demographics

Of the 11,031 patients, 10,009 (90.7%) had an average adherence calculable, 3,329 (30.2%) had ≥1 percentage change in non-HDL cholesterol result, and 9,440 (85.6%) had ≥1 LDL cholesterol result. Over an average 4.5 years follow-up, the mean-average statin adherence was 79.6%, and 84.5% of patients achieved ≥50% average statin adherence and 68.9% had ≥80%. Three-quarters of patients had LDL≤1.8mmol/L and half achieved ≥40% reduction in non-HDL at least once during follow-up.

Table 1 shows demographics by lipid target achievement and average adherence. Males were more likely to be adherent and achieve lipid targets. Patients who achieved ≥40% reduction in non-HDL and patients who were adherent (at either threshold) were younger on average. Those residing in more deprived areas were more likely to have adherence ≥50%, but this was less evident for ≥80%, and those in the most and least deprived areas were more likely to meet the LDL target. Patients with a diagnosis of diabetes were more likely to achieve LDL≤1.8mmol/l but were less likely to meet the non-HDL target. However, patients diagnosed with Type 2 diabetes were more likely to be adherent, particularly ≥50%, whilst patients diagnosed with Type 1 diabetes were less likely. Finally, patients with prior MIs were slightly more likely to achieve LDL≤1.8mmol/L and have adherence ≥50%, but the inverse was true for the non-HDL target.

Adherence and Lipid Targets

Adherence was associated with lipid target achievement (Tables s1-2). Those with adherence <50% had an increased risk of not meeting LDL≤1.8mmol/L in unadjusted (OR 1.99 (95% CI 1.75, 2.26), p<0.0001) and adjusted models (OR 2.03 (1.78, 2.31), p<0.0001), and had an adjusted odds ratio of not meeting the non-HDL target of 4.5 (95% CI: 3.62, 5.54, p<0.0001). Similar patterns were observed for adherence <80%. A 10% decrease in adherence was associated with a 20% increase in odds of not meeting the non-HDL target, and a 10% increase in odds of not achieving LDL≤1.8mmol/L, with both increases unaltered following adjustment. In a sensitivity analysis including incomplete years of follow-up, odds ratios were slightly lower but retained statistical significance (Tables s3-4).

Associations with All-Cause and Cardiovascular Mortality

For peer review only - http://bmjopen.bmj.com/site/about/guidelines.xhtml

All adherence and lipid target exposures were associated with all-cause and CVD mortality. For all-cause mortality in unadjusted models, non-achievement of LDL≤1.8mmol/L and the non-HDL target was associated with 1.3 times and 2.2 times greater risks, respectively (Figure 2a, Table s5). Following adjustment, this risk remained similar for LDL≤1.8mmol/L but attenuated to 1.8 times higher for the non-HDL target. The hazard ratios were nearly identical for CVD mortality, although fewer events resulted in wider confidence intervals (Figure 2b, Table s5).

For average adherence, similar effect sizes were reported for both all-cause and CVD mortality (Figure 2, Tables s6-7). In unadjusted analyses, adherence <50% was associated with a hazard of all-cause mortality 1.6 times, and adherence <80% with a hazard 1.5 times, that of those above each threshold. A 10% decrease in adherence was associated with 7% and 6% increases in the risk of all-cause and CVD mortality respectively, which reduced to 3% in adjusted analyses. For the 50% and 80% thresholds, adjustment attenuated the hazard ratios to 1.2 and 1.4, respectively. In a sensitivity analysis including incomplete years of follow-up in the average adherence calculation, the unadjusted hazard ratios were slightly larger, increasing to two times higher risk for all-cause mortality, and 1.8 times higher for CVD mortality (Figure s1, Tables s8-9). When adjusted, this attenuated to hazard ratios of 1.5 for <50% and 1.7 for <80% adherence for all-cause mortality, and 1.4 and 1.5 for CVD mortality for <50% and <80% adherence respectively.

Associations with Recurrent Myocardial Infarction

In both unadjusted and adjusted models, there were no associations between the achievement of LDL≤1.8mmol/l, or statin adherence, and recurrent MIs during follow-up (Figure 3, Tables s10-11). Nevertheless, there was an association with the non-HDL target; non-achievement was associated with 21% higher odds of recurrent MI in unadjusted analyses. For all models, adjustment had minimal impact on the odds ratios. In a sensitivity analysis (Figure s2, Table s12), where average adherence included incomplete years, these associations remained consistent.

Discussion

In this Scottish secondary prevention population, 76% achieved LDL≤1.8mmol/L, 51% achieved ≥40% reduction in non-HDL, and 85% and 69% had an average statin adherence ≥50% and ≥80% respectively. Lower average statin adherence, and failure to achieve guideline-recommended lipid targets, were strongly associated with higher risks of all-cause and CVD mortality and were significantly associated with each other. In adjusted models, those with adherence <50% were 24%

For peer review only - http://bmjopen.bmj.com/site/about/guidelines.xhtml

more likely to die, and those not achieving LDL≤1.8mmol/L were 32% more likely to die, with similar patterns observed when cardiovascular causes were considered.

This study advances the literature by the simultaneous comparison of both adherence and lipid targets as surrogates of statin effectiveness, showing these measurements have similar associations with outcomes. However, guidelines do not currently recommend the use of adherence data in estimating the clinical effectiveness of statins in patients. Our data support the notion that a patient's statin adherence, estimated using MPR, may be a useful risk surrogate. Additionally, in this analysis, a 50% threshold appears to have approximately similar associations with outcomes as the, conventionally used, 80% threshold. Given that alternate-day statin dosing is a common approach in routine clinical practice where daily statins are not tolerated [16], the 50% threshold, therefore, seems pragmatic in this instance. Furthermore, these data also suggest that where adherence data are readily available, annual lipid tests may not be necessary for CVD risk assessment in secondary prevention, potentially reducing costs and workload for health services. However, this hypothesis requires further research.

Lowering LDL has consistently demonstrated significant reductions in mortality within those with established disease [1]. Consistent with this, we report associations of LDL and non-HDL targets with all-cause and CVD specific mortality outcomes. However, whilst the LDL≤1.8mmol/l target used was the one that was recommended by the ESC at the time of data collection, this has since been revised in the latest version to ≤1.4mmol/l in secondary prevention and ≤1.0mmol/l after multiple cardiovascular events [24]. This may limit the applicability of these results in future cohorts, although LDL≤1.8mmol/l still remains a commonly used target in many other guidelines [14]. Furthermore, the stronger associations with the non-HDL target than the associations with the LDL target are likely to have arisen due to the nature of the targets e.g. percentage change vs absolute value, rather than the lipid profile components themselves. For example, associations between absolute values of non-HDL and LDL with a composite endpoint of cardiovascular events are approximately equivalent [25]. Nevertheless, percentage change targets within secondary prevention could prove problematic, with many patients unlikely to suitable baselines for the calculation to be performed. This was evident here where percentage change could only be calculated for 30% of the cohort.

The consistent associations we report between adherence and mortality are also broadly similar to findings observed in other cohorts [1,7]. In high-risk patients in a UK primary care cohort, adherence

For peer review only - http://bmjopen.bmj.com/site/about/guidelines.xhtml

<80% was associated with a 50% greater hazard of all cardiovascular events following adjustment for demographic factors and comorbidities [7]. Our data suggest that a 50% adherence threshold had a similar association in unadjusted models, although this was attenuated after adjustment. Furthermore, this was also lower than associations observed in a meta-analysis of 12 studies (n=~1m) who found that patients (in primary and secondary prevention) with poor adherence (<80%) had an 82% greater risk of all-cause mortality. However, although the definition for adherent was harmonised to the 80% threshold to facilitate the meta-analysis, the methods used for determining whether a patient was adherent differed between the included studies, and adjustment for confounders was not consistent [26].

In this study there was no association between average statin adherence nor the achievement of lipid targets with recurrent MIs. This second observation is consistent with other observational cohorts where post MI LDL demonstrated poor predictive performance for recurrent ASCVD events [27,28]. However, statin non-adherence has previously demonstrated a stronger, and intensity-dependent, association with further events in a cohort study in Finland between 2004 and 2016 (n=28,625) [28]. One explanation for this could be that in our analysis, average statin adherence and lipid target achievement was determined from information collected before and after recurrent MIs, whereas Lassenius et al (2020) used only data for the intervening time [28]. Furthermore, as many patients in our analysis experienced MIs early in follow-up, these exposures are more likely to reflect patient behaviour following multiple MIs, rather than the intervening time. For example, patients may initially increase their adherence or treatment intensity or only achieve lipid targets, after further MIs [10,28]. However, one analysis found only 5% more patients achieved LDL≤1.8mmol/L and 7% increased their treatment intensity after their second event [10].

The associations between statin adherence and lipid target achievement also expand on associations with mortality, with similar associations reported in other observational cohorts using electronic prescribing records. For example, in Georgia, USA (n=~1,000), those with adherence <50%, as measured using proportion of days covered (PDC) from dispensing data, were at 88% greater risk of not achieving a 30% reduction in LDL [11]. Similarly, in Greece, another study of patients with established CVD found that those with adherence<80%, as measured with PDC and following adjustment, were 91% more likely to achieve an LDL≤1.8mmol/l after one year than those with poor adherence. However, despite this, not all patients who were considered to have good adherence achieved the targeted LDL value from the guidelines [29]. This was also observed in an analysis of UK primary care data utilising prescribing data, following a cardiovascular event, approximately 70% of

For peer review only - http://bmjopen.bmj.com/site/about/guidelines.xhtml

patients achieved ≥80% statin adherence (as measured by PDC) in the first year, despite a higher percentage failing to have LDL≤1.8mmol/l [10]. Therefore, whilst adherence appears to be associated with cholesterol levels, it is not the only contributing factor. Likewise, this pattern is observed in this cohort, which calculated MPR, a similar and widely used method, on dispensing data. Nevertheless, a temporal relationship between statin adherence and lipid target achievement cannot be established, in these analyses. Both were captured simultaneously during follow-up and therefore, whilst an association between the two can be observed, it is difficult to discern, at the population level, its direction.

Strengths and Limitations

This is a contemporary, large dataset from a real-world population. The generalisability of these findings, particularly to the UK population, is therefore good. In this analysis, we confirm and expand on previous research by illustrating that lipid levels and statin adherence are important predictors of death and increase the duration of follow-up beyond the initial few years following statin initiation. This facilitated the estimation of longer-term average statin adherence and more time for target lipid levels to be achieved. Furthermore, the adherence measure used is arguably stronger evidence of medication use due to prescriptions being encashed [30], rather than prescribed. However, as with all indirect measures of adherence, possession of the medication is not sufficient to ensure patient compliance, and consequently patient adherence may have been overestimated.

In models using average adherence, only those who survived ≥1 year of follow-up were included, reducing the potential for reverse causality. Indeed, in sensitivity analyses, effect sizes were larger for mortality and smaller for lipid target achievement where incomplete time windows were included. However, in all mortality models, time-window bias could have arisen due to differing lengths of follow-up [31]. Specifically, patients who died had reduced opportunities to achieve lipid targets and change their adherence behaviour. The construction of the cohort also meant that patients were assumed to be alive in the absence of a death record, and by allowing for at least three years of follow-up to be available (except in the instances of death), this could have resulted in a higher proportion of patients who had moved out of the region. Such patients may be more likely to have higher degrees of frailty or severity of illnesses, and this could have resulted in biases in both the frequency of patient outcomes and their behaviour.

Finally, adjustment for age, sex, deprivation, and year of MI, did not substantially alter the effect sizes or their significance. However, this adjustment was not comprehensive, and was limited by the

For peer review only - http://bmjopen.bmj.com/site/about/guidelines.xhtml

variables contained within the data extract. Indeed, many comorbidities and lifestyle factors which might influence both the exposures and the outcomes in these analyses were not routinely captured. Therefore, other confounders are likely to remain, although the primary aim of this study was to contrast the associations of lipid targets and adherence with outcomes, rather than make causal inferences. For example, adherence in secondary prevention is higher in those with a greater number of comorbidities [10,11], which may also increase the risk of further events. In this cohort, those experiencing further events were more likely to have a diagnosis of diabetes or had a prior MI; both of which have been associated with higher adherence [10,12]. Consequently, given the observational nature of these data, no causal conclusions are drawn.

Conclusions

A significant proportion of the post MI population achieves lipid targets and high average statin adherence, and both have similar strengths of association with reduced mortality. These associations, and those observed between lipid target achievement and adherence, are consistent with the evidence base for statins. We also broadly validate the existing Scottish SIGN non-HDL guidance as clinically relevant, however low coverage for percentage change variables in secondary prevention limits its practical use in these settings.

Funding

RB is funded as part of the Medical Research Council Doctoral Training Programme in Precision Medicine (MR/N013166/1). The Medical Research Council has had no role in any aspect of this work.

Competing Interests

RB, JLewsey, SW, and JLogue declare no conflict. PW has received grant support from Roche Diagnostics, AstraZeneca, Novartis, and Boehringer Ingelheim.

Author's Contributions

JLogue and PW conceived and designed the study, and acquired the data. RB conducted statistical analyses and drafted the manuscript. PW checked and validated statistical analyses. JLewsey, and SW contributed to the analytical design and interpretation of the data. JLewsey, SW, JLogue and PW critically revised the manuscript for important intellectual content. RB, JLewsey, SW, JLogue and PW all gave final approval for publication. PW is guarantor and agrees to be accountable for all aspects of work ensuring integrity and accuracy.

For peer review only - http://bmjopen.bmj.com/site/about/guidelines.xhtml

For peer review only

Ethical Approval Information

This study used fully anonymised health record data accessed via a trusted research environment and was therefore exempt from ethical review. Permission was given by NHS GGC Safe Haven local Privacy Advisory Committee (membership includes the ethics committee chair) (project GSH/17/CA/012 PMCVD).

Data Availability Statement

Deidentified data may be obtained by bona fide researchers, for the purposes of research in the public interest, from NHS GGC Safe Haven, via an approval process (https://www.nhsggc.org.uk/about-us/professional-support-sites/glasgow-safe-haven/). Code files are available on request (Rosemary.Brown@glasgow.ac.uk).

For peer review only

For peer review only - http://bmjopen.bmj.com/site/about/guidelines.xhtml

References

1 Cholesterol Treatment Trialists' (CTT) Collaboration. Efficacy and safety of more intensive lowering of LDL cholesterol: a meta-analysis of data from 170 000 participants in 26 randomised trials. *Lancet* 2010;**376**:1670–81. doi:10.1016/S0140-6736(10)61350-5

2 World Health Organization. Cardiovascular Diseases (CVDs): Fact Sheet. 2017.https://www.who.int/en/news-room/fact-sheets/detail/cardiovascular-diseases-(cvds) (accessed 3 Oct 2019).

3 NICE. Cardiovascular disease: risk assessment and reduction, including lipid modification (CG181). 2014.

4 SIGN. SIGN 149: Risk estimation and the prevention of cardiovascular disease. Published Online First: 2017.http://www.sign.ac.uk/assets/sign149.pdf

5 Cholesterol Treatment Trialists' (CTT) Collaborators. The effects of lowering LDL cholesterol with statin therapy in people at low risk of vascular disease: meta-analysis of individual data from 27 randomised trials. *Lancet* 2012;**380**:581–90. doi:10.1016/S0140-6736(12)60367-5

6 Herrett E, Gallagher AM, Bhaskaran K, *et al.* Data Resource Profile: Clinical Practice Research Datalink (CPRD). *Int J Epidemiol* 2015;**44**:827–36. doi:10.1093/ije/dyv098

7 Khunti K, Danese MD, Kutikova L, *et al.* Association of a Combined Measure of Adherence and Treatment Intensity With Cardiovascular Outcomes in Patients With Atherosclerosis or Other Cardiovascular Risk Factors Treated With Statins and/or Ezetimibe. *JAMA Netw Open* 2018;**1**. doi:10.1001/jamanetworkopen.2018.5554

8 Naderi SH, Bestwick JP, Wald DS. Adherence to drugs that prevent cardiovascular disease: Meta-analysis on 376,162 patients. *Am J Med* 2012;**125**:882-887.e1. doi:10.1016/j.amjmed.2011.12.013

9 Nordstrom BL, Collins JM, Donaldson R, *et al.* Treatment patterns and lipid levels among patients with high-risk atherosclerotic CVD in the UK. *Br J Cardiol* Published Online First: 2015. doi:10.5837/bjc.2015.034

10 Danese MD, Gleeson M, Kutikova L, *et al.* Management of lipid-lowering therapy in patients with cardiovascular events in the UK: A retrospective cohort study. *BMJ Open* 2017;**7**:1–10. doi:10.1136/bmjopen-2016-013851

11 Vupputuri S, Joski PJ, Kilpatrick R, *et al.* LDL cholesterol response and statin adherence among high-risk patients initiating treatment. *J Manag Care* 2016;**22**.https://www.ajmc.com/journals/issue/2016/2016-vol22-n3/ldl-cholesterol-response-and-statin-adherence-among-high-risk-patients-initiating-treatment

12 Hope HF, Binkley GM, Fenton S, *et al.* Systematic review of the predictors of statin adherence for the primary prevention of cardiovascular disease. *PLoS One* 2019;**14**. doi:10.1371/journal.pone.0201196

13 Chen ST, Huang ST, Shau WY, *et al.* Long-term statin adherence in patients after hospital discharge for new onset of atherosclerotic cardiovascular disease: A population-based study of real world prescriptions in Taiwan. *BMC Cardiovasc Disord* 2019;**19**. doi:10.1186/s12872-019-1032-4

14 Brown RE, Welsh P, Logue J. Systematic review of clinical guidelines for lipid lowering in the secondary prevention of cardiovascular disease events. *Open Hear* 2020;**7**:e001396. doi:10.1136/openhrt-2020-001396

15 Raebel MA, Schmittdiel J, Karter AJ, *et al.* Standardizing Terminology and Definitions of Medication Adherence and Persistence in Research employing Electronic Databases. *Med Care* 2014;**51**:11–21. doi:10.1097/MLR.0b013e31829b1d2a.Standardizing

16 Jordanov MS, Abou Assi H. Statins. In: Yassine H, ed. *Lipid Management: From Basics to Clinic*. Springer International Publishing 2015. 123–47. doi:10.1007/978-3-319-11161-2_8

17 Karve S, Cleves MA, Helm M, *et al.* Good and poor adherence: optimal cut-point for adherence measures using administrative claims data. *Curr Med Res Opin* 2009;**25**:2303–10. doi:10.1185/03007990903126833

For peer review only - http://bmjopen.bmj.com/site/about/guidelines.xhtml

18 Piepoli MF, Hoes AW, Agewall S, *et al.* 2016 European Guidelines on cardiovascular disease prevention in clinical practice. *Eur Heart J* 2016;**37**:2315–81. doi:10.1093/eurheartj/ehw106
19 NHS Greater Glasgow and Clyde. NHS GGC Director of Public Health Report 2015-2017; Chapter 5. 2015. https://www.nhsggc.org.uk/media/234515/nhsggc_ph_dphreport2015_health_related_behaviours.pdf
20 R Core Team. R: A Language and Environment for Statistical Computing. 2018.http://www.r-project.org/
21 Gordon M, Lumley T. forestplot: Advanced Forest Plot Using 'grid' Graphics in R. 2020.https://cran.r-project.org/web/packages/forestplot/index.html (accessed 15 Jan 2021).
22 Therneau TM. A Package for Survival Analysis in R. 2020.https://cran.r-project.org/package=survival
23 Kassambara A, Kosinski M, Biecek P, *et al.* Drawing Survival Curves using 'ggplot2'. 2020.https://cran.r-project.org/package=survminer
24 Mach F, Baigent C, Catapano AL, *et al.* 2019 ESC/EAS Guidelines for the management of dyslipidaemias: Lipid modification to reduce cardiovascular risk. *Eur Heart J* 2020;**41**:111–88. doi:10.1093/eurheartj/ehz455
25 Welsh C, Celis-Morales CA, Brown R, *et al.* Comparison of Conventional Lipoprotein Tests and Apolipoproteins in the Prediction of Cardiovascular Disease: Data from UK Biobank. *Circulation* Published Online First: 2019. doi:10.1161/CIRCULATIONAHA.119.041149
26 Chowdhury R, Khan H, Heydon E, *et al.* Adherence to cardiovascular therapy: A meta-analysis of prevalence and clinical consequences. *Eur Heart J* 2013;**34**:2940–8. doi:10.1093/eurheartj/eht295
27 Ohm J, Hjemdahl P, Skoglund PH, *et al.* Lipid levels achieved after a first myocardial infarction and the prediction of recurrent atherosclerotic cardiovascular disease. *Int J Cardiol* 2019;**296**:1–7. doi:10.1016/j.ijcard.2019.07.001
28 Lassenius MI, Toppila I, Bergius S, *et al.* Cardiovascular event rates increase after each recurrence and associate with poor statin adherence. *Eur J Prev Cardiol* 2020;:2047487320904334. doi:10.1177/2047487320904334
29 Zafeiropoulos S, Farmakis I, Kartas A, *et al.* Reinforcing adherence to lipid-lowering therapy after an acute coronary syndrome: A pragmatic randomized controlled trial. *Atherosclerosis* 2021;**323**:37–43. doi:10.1016/J.ATHEROSCLEROSIS.2021.03.013
30 Information Services Division. Prescribing Information System Fields Document. Prescr. Inf. Syst. Fields (1993 - Present. 2010;:18.https://www.isdscotland.org/Health-Topics/Prescribing-and-Medicines/Prescribing-Datamarts/docs/PIS_fields_for_researchers_v5_eDRIS Guidance.pdf (accessed 3 May 2018).
31 Di Martino M, Kirchmayer U, Agabiti N, *et al.* The impact of time-window bias on the assessment of the long-term effect of medication adherence: The case of secondary prevention after myocardial infarction. *BMJ Open* 2015;**5**:e007866. doi:10.1136/bmjopen-2015-007866

For peer review only - http://bmjopen.bmj.com/site/about/guidelines.xhtml

Figure Legends

Figure 1: Derivation of the Post Myocardial Infarction Population; MI = Myocardial Infarction, SIMD = Scottish Index of Multiple Deprivation.

Figure 2: Hazard Ratios of A) All-Cause and B) CVD Mortality by Average Statin Adherence and Achievement of Plasma Lipid Targets. Ratios presented for unadjusted (solid line and square) and adjusted (dashed line and circle) analyses (adjusted for age, sex, year of MI, and SIMD quintile). CVD death defined by ICD10 I00-I99 as the underlying cause. Average adherence calculated as the patient's mean-average annual medication possession ratio of complete years of follow-up. Non-HDL target: ≥40% reduction from a pre-MI baseline, LDL target: ≤1.8mmol/l. Patients classified as meeting a plasma lipid target if they achieved the target in any year of their follow-up, and not meeting a target otherwise. Cts = Continuous; CI = Confidence Interval.

Figure 3: Odds of MIs During Follow-Up by Average Statin Adherence and Achievement of Plasma Lipid Targets (from NICE and ESC Recommendations). Ratios presented for unadjusted (solid line and square) and adjusted (dashed line and circle) analyses (adjusted for age, sex, year of MI, and SIMD quintile). CVD death defined by ICD10 I00-I99 as the underlying cause. Models fitted using available case analysis. Average adherence calculated as the patient's mean-average annual medication possession ratio of complete years of follow-up. Non-HDL target: ≥40% reduction from a pre-MI baseline, LDL target: ≤1.8mmol/l. Patients classified as meeting a plasma lipid target if they achieved the target in any year of their follow-up, and not meeting a target otherwise. Cts = Continuous; CI = Confidence Interval.

For peer review only

For peer review only - http://bmjopen.bmj.com/site/about/guidelines.xhtml

Table 1: Demographics by Average Statin Adherence (50% and 80%) and Achievement of Lipid Targets

Target	Average Adherence ≥50%		Average Adherence ≥80%		≥40% reduction non-HDL*		LDL ≤1.8mmol/l	
	**Met**<br>8,461 (84.5%)	**Not Met**<br>1,548 (15.5%)	**Met**<br>6,894 (68.9%)	**Not Met**<br>3,115 (31.1%)	**Met**<br>1,709 (51.3%)	**Not Met**<br>1,620 (48.7%)	**Met**<br>7,171 (76.0%)	**Not Met**<br>2,269 (24.0%)
**Gender**								
Male	5,337 (63.1%)	829 (53.6%)	4,368 (63.4%)	1,798 (57.7%)	1,102 (64.5%)	911 (56.2%)	4,566 (63.7%)	1,342 (59.1%)
Female	3,124 (36.9%)	719 (46.4%)	2,526 (36.6%)	1,317 (42.3%)	607 (35.5%)	709 (43.8%)	2,605 (36.3%)	927 (40.9%)
**Age at MI (years)**								
Mean (SD)	65.3 (13.2)	68.1 (15.9)	65.4 (13.0)	66.4 (15.2)	63.6 (12.6)	67.0 (13.5)	65.4 (13.2)	65.5 (13.9)
**SIMD 2012 Quintile (NHS GGC)**								
1 (Most)	2,058 (24.3%)	347 (22.4%)	1,652 (24.0%)	753 (24.2%)	398 (23.3%)	381 (23.5%)	1,824 (25.4%)	412 (18.2%)
2	1,979 (23.4%)	318 (20.5%)	1,625 (23.6%)	672 (21.6%)	389 (22.8%)	363 (22.4%)	1,620 (22.6%)	551 (24.3%)
3	1,750 (20.7%)	338 (21.8%)	1,389 (20.1%)	699 (22.4%)	349 (20.4%)	349 (21.5%)	1,438 (20.1%)	535 (23.6%)
4	1,395 (16.5%)	289 (18.7%)	1,153 (16.7%)	531 (17.0%)	284 (16.6%)	283 (17.5%)	1,140 (15.9%)	454 (20.0%)
5 (Least)	1,279 (15.1%)	256 (16.5%)	1,075 (15.6%)	460 (14.8%)	289 (16.9%)	244 (15.1%)	1,149 (16.0%)	317 (14.0%)
**Diabetes at MI**	1,535 (18.1%)	258 (16.7%)	1,249 (18.1%)	544 (17.5%)	285 (16.7%)	369 (22.8%)	1,436 (20.0%)	312 (13.8%)
Type 1	80 (0.9%)	26 (1.7%)	63 (0.9%)	43 (1.4%)	12 (0.7%)	29 (1.8%)	73 (1.0%)	32 (1.4%)
Type 2	1,455 (17.2%)	232 (15.0%)	1,186 (17.2%)	501 (16.1%)	273 (16.0%)	340 (21.0%)	1,363 (19.0%)	280 (12.3%)
**Prior MI**	529 (6.3%)	85 (5.5%)	425 (6.2%)	189 (6.1%)	34 (2.0%)	98 (6.0%)	471 (6.6%)	114 (5.0%)
1	412 (4.9%)	68 (4.4%)	330 (4.8%)	150 (4.8%)	28 (1.6%)	79 (4.9%)	359 (5.0%)	94 (4.1%)
>1	117 (1.4%)	17 (1.1%)	95 (1.4%)	39 (1.3%)	6 (0.4%)	19 (1.2%)	112 (1.6%)	20 (0.9%)

***refers to the percentage reduction in non-HDL cholesterol from the pre-MI baseline. MI = Myocardial Infarction; SD = Standard Deviation; IQR = Interquartile Range, SIMD = Scottish Index of Multiple Deprivation. Numbers are N (%) unless otherwise specified. Percentages are calculated within columns, except for the header where percentages are calculated from total with information. For adherence, patients were only included if ≥1 complete year of follow-up was available. For lipid targets, patients were included if a necessary test result was available.**

For peer review only

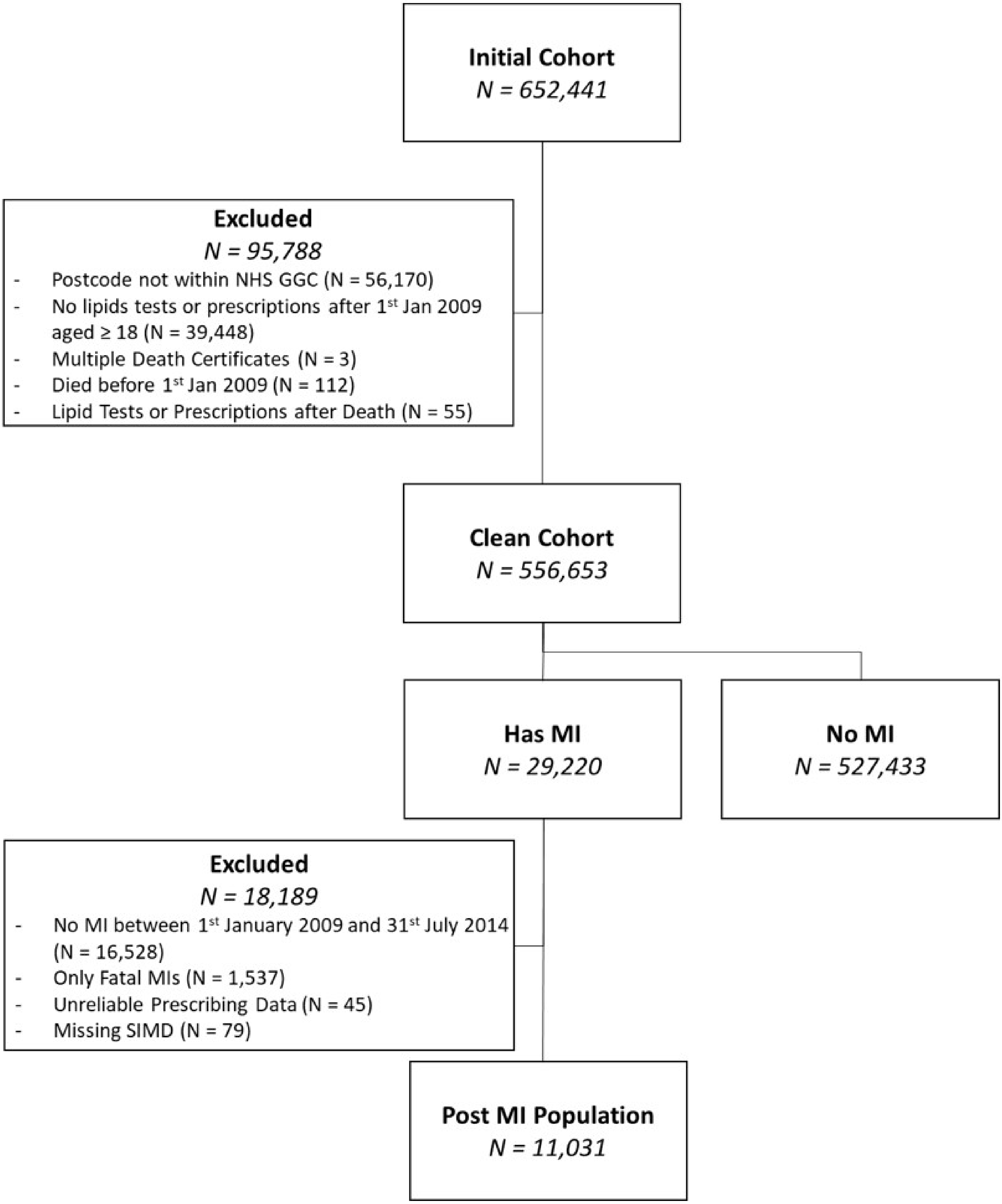
Initial Cohort
N = 652,441
Excluded
N = 95,788
- Postcode not within NHS GGC (N = 56,170)
- No lipids tests or prescriptions after 1st Jan 2009 aged ≥ 18 (N = 39,448)
- Multiple Death Certificates (N = 3)
- Died before 1st Jan 2009 (N = 112)
- Lipid Tests or Prescriptions after Death (N = 55)
Clean Cohort
N = 556,653
Has MI
N = 29,220
No MI
N = 527,433
Excluded
N = 18,189
- No MI between 1st January 2009 and 31st July 2014 (N = 16,528)
- Only Fatal MIs (N = 1,537)
- Unreliable Prescribing Data (N = 45)
- Missing SIMD (N = 79)
Post MI Population
N = 11,031

A – Overall Mortality

	Events	N	Hazard Ratio (95% CI)	p−value
**Average Statin Adherence (Cts)**				
10% Decrease	2726	10009	1.07 (1.06 to 1.08)	<0.0001
	2726	10009	1.03 (1.02 to 1.05)	<0.0001
**Average Statin Adherence (50%)**				
Achieved	2162	8461	1.00	
Not Achieved	564	1548	1.58 (1.44 to 1.74)	<0.0001
	564	1548	1.24 (1.13 to 1.37)	<0.0001
**Average Statin Adherence (80%)**				
Achieved	1656	6894	1.00	
Not Achieved	1070	3115	1.53 (1.42 to 1.66)	<0.0001
	1070	3115	1.37 (1.26 to 1.48)	<0.0001
**Non−HDL Target**				
Achieved	286	1709	1.00	
Not Achieved	516	1620	2.18 (1.87 to 2.49)	<0.0001
	516	1620	1.76 (1.52 to 2.03)	<0.0001
**LDL Target**				
Achieved	1886	7171	1.00	
Not Achieved	658	2269	1.27 (1.16 to 1.39)	<0.0001
	658	2269	1.32 (1.20 to 1.44)	<0.0001

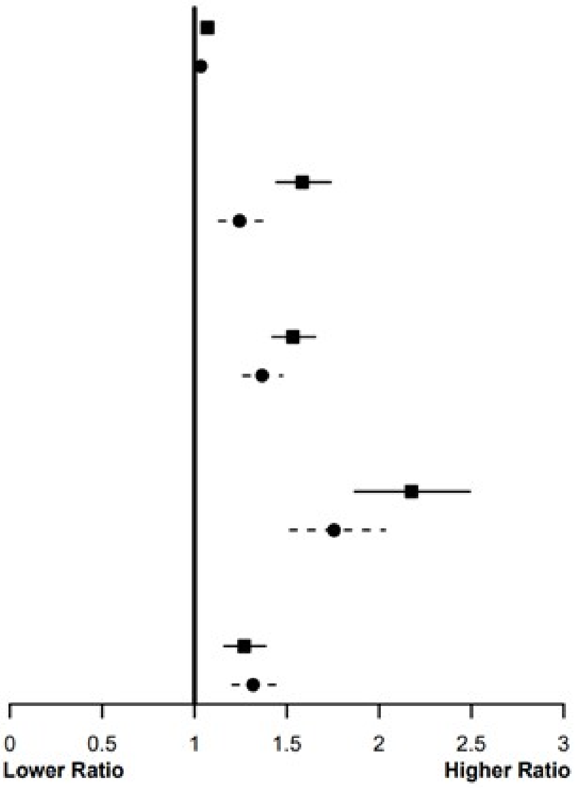

B – CVD Mortality

	Events	N	Hazard Ratio (95% CI)	p−value
**Average Statin Adherence (Cts)**				
10% Decrease	894	10009	1.06 (1.04 to 1.09)	<0.0001
	894	10009	1.03 (1.01 to 1.05)	0.0072
**Average Statin Adherence (50%)**				
Achieved	708	8461	1.00	
Not Achieved	186	1548	1.60 (1.36 to 1.88)	<0.0001
	186	1548	1.24 (1.05 to 1.46)	0.0116
**Average Statin Adherence (80%)**				
Achieved	558	6894	1.00	
Not Achieved	336	3115	1.43 (1.25 to 1.64)	<0.0001
	336	3115	1.29 (1.12 to 1.48)	0.0003
**Non−HDL Target**				
Achieved	94	1709	1.00	
Not Achieved	175	1620	2.21 (1.72 to 2.85)	<0.0001
	175	1620	1.82 (1.41 to 2.34)	<0.0001
**LDL Target**				
Achieved	629	7171	1.00	
Not Achieved	222	2269	1.29 (1.11 to 1.51)	0.0013
	222	2269	1.30 (1.11 to 1.52)	0.0012

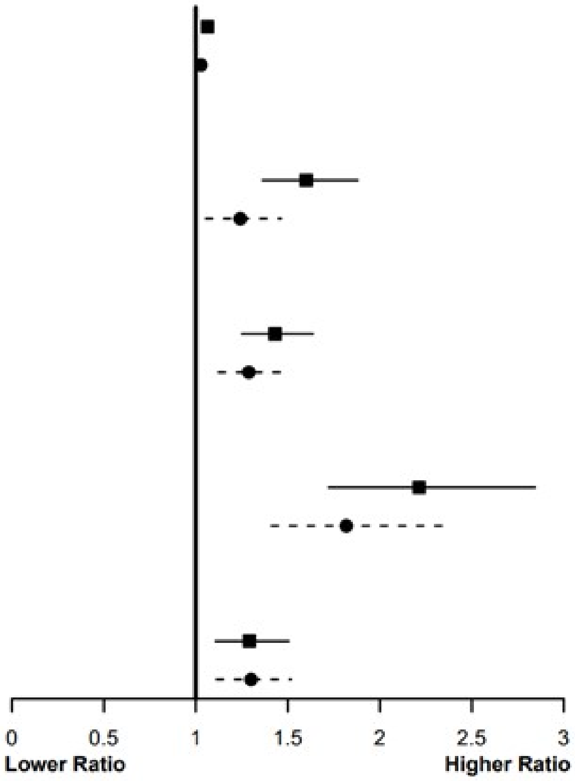

	Events	N	Odds Ratio (95% CI)	p−value
**Average Statin Adherence (Cts)**				
10% Decrease	2918	10009	1.00 (0.98 to 1.01)	0.5141
	2918	10009	0.99 (0.98 to 1.01)	0.4278
**Average Statin Adherence (50%)**				
Achieved	2492	8461	1.00	
Not Achieved	426	1548	0.91 (0.81 to 1.03)	0.1221
	426	1548	0.90 (0.79 to 1.01)	0.0803
**Average Statin Adherence (80%)**				
Achieved	1978	6894	1.00	
Not Achieved	940	3115	1.07 (0.98 to 1.18)	0.1309
	940	3115	1.07 (0.98 to 1.18)	0.1346
**Non−HDL Target**				
Achieved	490	1709	1.00	
Not Achieved	529	1620	1.21 (1.04 to 1.40)	0.0127
	529	1620	1.20 (1.03 to 1.40)	0.0161
**LDL Target**				
Achieved	2159	7171	1.00	
Not Achieved	687	2269	1.01 (0.91 to 1.12)	0.8776
	687	2269	1.00 (0.90 to 1.12)	0.9419

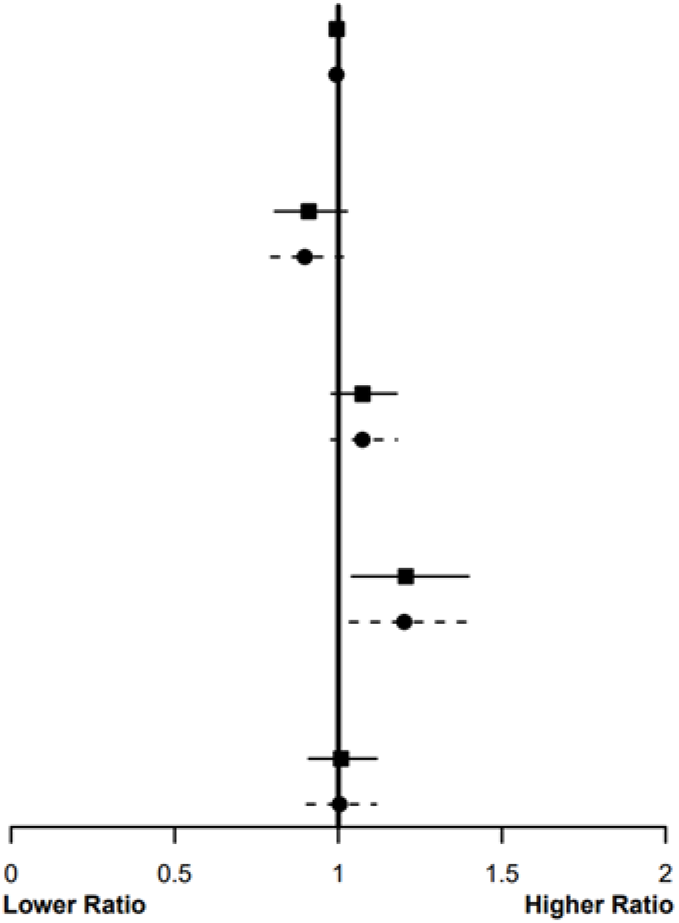

Supplementary Material

For peer review only

Table s1: Models for Non-HDL Target by Statin Adherence Thresholds

Model/Variable	Continuous Adherence		50% Adherence		80% Adherence	
	Odds Ratio (95% CI)	p-value	Odds Ratio (95% CI)	p-value	Odds Ratio (95% CI)	p-value
Unadjusted Model						
**Adherence Target**						
Achieved (ref.)	--		1.00	<0.0001	1.00	<0.0001
Not Achieved	--		4.77 (3.87, 5.90)		3.03 (2.60, 3.54)	
10% Decrease	1.24 (1.20, 1.27)	<0.0001				
Adjusted Model						
**Adherence Target**						
Achieved (ref.)	--		1.00	<0.0001	1.00	<0.0001
Not Achieved	--		4.47 (3.62, 5.54)		2.98 (2.55, 3.48)	
10% Decrease	1.23 (1.20, 1.26)	<0.0001				

Table s2: Models for LDL Target by Statin Adherence Thresholds

Model/Variable	Continuous Adherence		50% Adherence		80% Adherence	
	Odds Ratio (95% CI)	p-value	Odds Ratio (95% CI)	p-value	Odds Ratio (95% CI)	p-value
Unadjusted Model						
**Adherence Target**						
Achieved (ref.)	--		1.00	<0.0001	1.00	<0.0001
Not Achieved	--		1.99 (1.75, 2.26)		1.62 (1.46, 1.80)	
10% Decrease	1.10 (1.08, 1.12)	<0.0001				
Adjusted Model						
**Adherence Target**						
Achieved (ref.)	--		1.00	<0.0001	1.00	<0.0001
Not Achieved	--		2.03 (1.78, 2.31)		1.70 (1.53, 1.88)	
10% Decrease	1.11 (1.09, 1.12)	<0.0001				

For peer review only

Table s3: Models for Non-HDL Target by Statin Adherence Thresholds (sensitivity inc. incomplete years of follow-up)

	Continuous Adherence		50% Adherence		80% Adherence	
**Model/Variable**	**Odds Ratio (95% CI)**	**p-value**	**Odds Ratio (95% CI)**	**p-value**	**Odds Ratio (95% CI)**	**p-value**
Unadjusted Model						
**Adherence Target**						
Achieved (ref.)	--		1.00	<0.0001	1.00	<0.0001
Not Achieved	--		4.30 (3.53, 5.23)		2.85 (2.46, 3.30)	
10% Decrease	1.23 (1.20, 1.26)	<0.0001				
Adjusted Model						
**Adherence Target**						
Achieved (ref.)	--		1.00	<0.0001	1.00	<0.0001
Not Achieved	--		3.98 (3.26, 4.85)		2.75 (2.37, 3.19)	
10% Decrease	1.22 (1.19, 1.25)	<0.0001				

Table s4: Models for LDL Target by Statin Adherence Thresholds (sensitivity inc. incomplete years of follow-up)

	Continuous Adherence		<50% Adherence		<80% Adherence	
**Model/Variable**	**Odds Ratio (95% CI)**	**p-value**	**Odds Ratio (95% CI)**	**p-value**	**Odds Ratio (95% CI)**	**p-value**
Unadjusted Model						
**Adherence Target**						
Achieved (ref.)	--		1.00	<0.0001	1.00	<0.0001
Not Achieved	--		1.86 (1.65, 2.09)		1.53 (1.39, 1.69)	
10% Decrease	1.09 (1.07, 1.10)	<0.0001				
Adjusted Model						
**Adherence Target**						
Achieved (ref.)	--		1.00	<0.0001	1.00	<0.0001
Not Achieved	--		1.90 (1.68, 2.14)		1.59 (1.44, 1.76)	
10% Decrease	1.09 (1.07, 1.11)	<0.0001				

For peer review only

Table s5: Models for All-Cause and CVD Mortality by Lipid Targets

Model/Variable	Non-HDL Target				LDL Target			
	All-Cause		Circulatory		All-Cause		Circulatory	
	Hazard Ratio (95% CI)	p-value	Hazard Ratio (95% CI)	p-value	Hazard Ratio (95% CI)	p-value	Hazard Ratio (95% CI)	p-value
Unadjusted Model								
**Lipid Target**								
Achieved (ref.)	1.00	<0.0001	1.00	<0.0001	1.00	<0.0001	1.00	0.0013
Not Achieved	2.16 (1.87, 2.49)		2.21 (1.72, 2.85)		1.27 (1.16, 1.39)		1.29 (1.11, 1.51)	
Adjusted Model								
**Lipid Target**								
Achieved (ref.)	1.00	<0.0001	1.00	<0.0001	1.00	<0.0001	1.00	0.0012
Not Achieved	1.76 (1.52, 2.03)		1.82 (1.41, 2.34)		1.32 (1.20, 1.44)		1.30 (1.11, 1.52)	

For peer review only

Table s6: Models for Overall Mortality by Statin Adherence Thresholds

Model/Variable	Continuous Adherence		50% Adherence		80% Adherence	
	Hazard Ratio (95% CI)	p-value	Hazard Ratio (95% CI)	p-value	Hazard Ratio (95% CI)	p-value
Unadjusted Model						
**Adherence Target**						
Achieved (ref.)	--		1.00	<0.0001	1.00	<0.0001
Not Achieved	--		1.58 (1.44, 1.73)		1.53 (1.42, 1.66)	
10% Decrease	1.07 (1.06, 1.08)	<0.0001				
Adjusted Model						
**Adherence Target**						
Achieved (ref.)	--		1.00	<0.0001	1.00	<0.0001
Not Achieved	--		1.24 (1.13, 1.37)		1.37 (1.26, 1.48)	
10% Decrease	1.03 (1.02, 1.05)	<0.0001				

Table s7: Models for CVD Mortality by Statin Adherence Thresholds

Model/Variable	Continuous Adherence		50% Adherence		80% Adherence	
	Hazard Ratio (95% CI)	p-value	Hazard Ratio (95% CI)	p-value	Hazard Ratio (95% CI)	p-value
Unadjusted Model						
**Adherence Target**						
Achieved (ref.)	--		1.00	<0.0001	1.00	<0.0001
Not Achieved	--		1.60 (1.36, 1.88)		1.43 (1.25, 1.64)	
10% Decrease	1.06 (1.04, 1.09)	<0.0001				
Adjusted Model						
**Adherence Target**						
Achieved (ref.)	--		1.00	0.0116	1.00	0.0003
Not Achieved	--		1.24 (1.05, 1.46)		1.29 (1.12, 1.48)	
10% Decrease	1.03 (1.01, 1.05)	0.0072				

For peer review only

A – Overall Mortality

	Events	N	Hazard Ratio (95% CI)	p−value
**Average Statin Adherence (Cts)**				
10% Decrease	3748	11031	1.10 (1.09 to 1.11)	<0.0001
	3748	11031	1.06 (1.05 to 1.07)	<0.0001
**Average Statin Adherence (50%)**				
Achieved	2715	8948	1.00	
Not Achieved	1033	2083	2.01 (1.87 to 2.16)	<0.0001
	1033	2083	1.49 (1.38 to 1.60)	<0.0001
**Average Statin Adherence (80%)**				
Achieved	1897	7053	1.00	
Not Achieved	1851	3978	2.05 (1.93 to 2.19)	<0.0001
	1851	3978	1.72 (1.61 to 1.83)	<0.0001
**Non−HDL Target**				
Achieved	286	1709	1.00	
Not Achieved	516	1620	2.18 (1.87 to 2.49)	<0.0001
	516	1620	1.76 (1.52 to 2.03)	<0.0001
**LDL Target**				
Achieved	1886	7171	1.00	
Not Achieved	658	2269	1.27 (1.16 to 1.39)	<0.0001
	658	2269	1.32 (1.20 to 1.44)	<0.0001

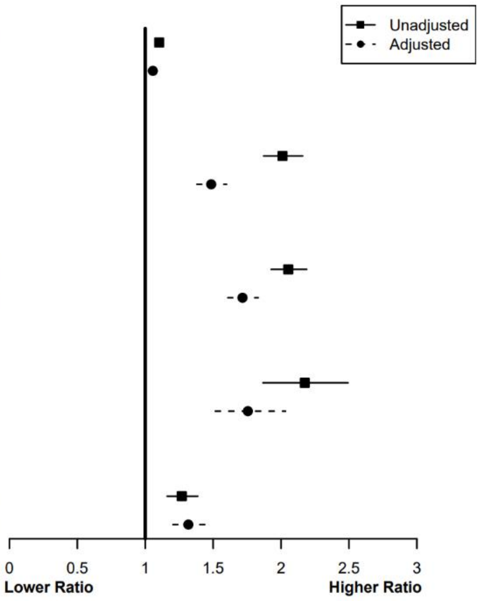

B – CVD Mortality

	Events	N	Hazard Ratio (95% CI)	p−value
**Average Statin Adherence (Cts)**				
10% Decrease	1200	11031	1.08 (1.07 to 1.10)	<0.0001
	1200	11031	1.04 (1.02 to 1.06)	<0.0001
**Average Statin Adherence (50%)**				
Achieved	891	8948	1.00	
Not Achieved	309	2083	1.84 (1.62 to 2.10)	<0.0001
	309	2083	1.35 (1.18 to 1.54)	<0.0001
**Average Statin Adherence (80%)**				
Achieved	646	7053	1.00	
Not Achieved	554	3978	1.81 (1.62 to 2.03)	<0.0001
	554	3978	1.53 (1.37 to 1.72)	<0.0001
**Non−HDL Target**				
Achieved	94	1709	1.00	
Not Achieved	175	1620	2.21 (1.72 to 2.85)	<0.0001
	175	1620	1.82 (1.41 to 2.34)	<0.0001
**LDL Target**				
Achieved	629	7171	1.00	
Not Achieved	222	2269	1.29 (1.11 to 1.51)	0.0013
	222	2269	1.30 (1.11 to 1.52)	0.0012

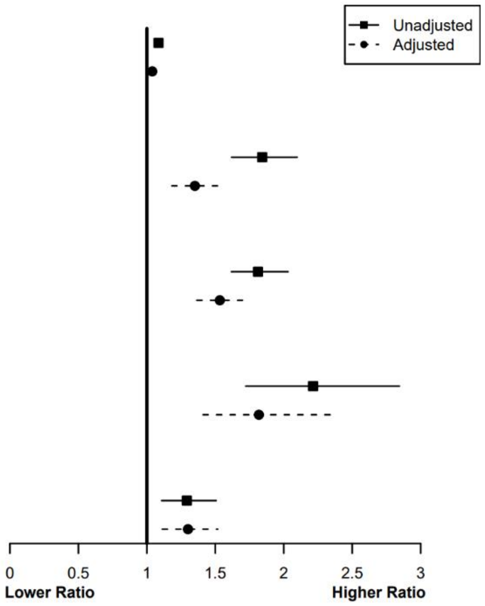

Figure s1: Hazard Ratios of A) All-Cause and B) CVD Mortality by Average Statin Adherence and Achievement of Plasma Lipid Targets (from NICE and ESC Recommendations) (Sensitivity inc. incomplete years of follow up)

Table s8: Models for Overall Mortality by Statin Adherence Thresholds (sensitivity inc. incomplete years of follow up)

	Continuous Adherence		50% Adherence		80% Adherence	
**Model/Variable**	**Hazard Ratio (95% CI)**	**p-value**	**Hazard Ratio (95% CI)**	**p-value**	**Hazard Ratio (95% CI)**	**p-value**
Unadjusted Model						
**Adherence Target**						
Achieved (ref.)	--		1.00	<0.0001	1.00	<0.0001
Not Achieved	--		2.01 (1.87, 2.16)		2.05 (1.93, 2.19)	
10% Decrease	1.10 (1.09, 1.11)	<0.0001				
Adjusted Model						
**Adherence Target**						
Achieved (ref.)	--		1.00	<0.0001	1.00	<0.0001
Not Achieved	--		1.49 (1.38, 1.60)		1.72 (1.61, 1.83)	
10% Decrease	1.06 (1.05, 1.07)	<0.0001				

Table s9: Models for CVD Mortality by Statin Adherence Thresholds (sensitivity inc. incomplete years of follow up)

	Continuous Adherence		50% Adherence		80% Adherence	
**Model/Variable**	**Hazard Ratio (95% CI)**	**p-value**	**Hazard Ratio (95% CI)**	**p-value**	**Hazard Ratio (95% CI)**	**p-value**
Unadjusted Model						
**Adherence Target**						
Achieved (ref.)	--		1.00	<0.0001	1.00	<0.0001
Not Achieved	--		1.84 (1.62, 2.10)		1.81 (1.62, 2.03)	
10% Decrease	1.09 (1.07, 1.10)	<0.0001				
Adjusted Model						
**Adherence Target**						
Achieved (ref.)	--		1.00	0.0001	1.00	<0.0001
Not Achieved	--		1.35 (1.18, 1.54)		1.53 (1.37, 1.72)	
10% Decrease	1.04 (1.02, 1.06)	<0.0001				

For peer review only

Table s10: Models for Recurrent MIs by Lipid Targets

Model/Variable	Non-HDL Target Odds Ratio (95% CI)	Non-HDL Target p-value	LDL Target Odds Ratio (95% CI)	LDL Target p-value
Unadjusted Model				
**Lipid Target**				
Achieved (ref.)	1.00	0.0127	1.00	0.8776
Not Achieved	1.21 (1.04, 1.40)		1.01 (0.91, 1.12)	
Adjusted Model				
**Lipid Target**				
Achieved (ref.)	1.00	0.0161	1.00	0.9419
Not Achieved	1.20 (1.03, 1.40)		1.00 (0.90, 1.12)	

For peer review only

Table s11: Models for Recurrent MIs by Statin Adherence Thresholds

	Continuous Adherence		50% Adherence		80% Adherence	
**Model/Variable**	**Odds Ratio (95% CI)**	**p-value**	**Odds Ratio (95% CI)**	**p-value**	**Odds Ratio (95% CI)**	**p-value**
Unadjusted Model						
**Adherence Target**						
Achieved (ref.)	--		1.00	0.1221	1.00	0.1309
Not Achieved	--		0.91 (0.81, 1.03)		1.07 (0.98, 1.18)	
10% Decrease	1.00 (0.98, 1.01)	0.5141				
Adjusted Model						
**Adherence Target**						
Achieved (ref.)	--		1.00	0.0803	1.00	0.1346
Not Achieved	--		0.90 (0.79, 1.01)		1.07 (0.98, 1.18)	
10% Decrease	0.99 (0.98, 1.01)	0.4278				

For peer review only

	Events	N	Odds Ratio (95% CI)	p−value
**Average Statin Adherence (Cts)**				
10% Decrease	3265	11031	0.99 (0.98 to 1.01)	0.3392
	3265	11031	0.99 (0.98 to 1.00)	0.2279
**Average Statin Adherence (50%)**				
Achieved	2684	8948	1.00	
Not Achieved	581	2083	0.90 (0.81 to 1.00)	0.0572
	581	2083	0.89 (0.80 to 0.99)	0.0255
**Average Statin Adherence (80%)**				
Achieved	2049	7053	1.00	
Not Achieved	1216	3978	1.08 (0.99 to 1.17)	0.1305
	1216	3978	1.07 (0.98 to 1.16)	0.1305
**Non−HDL Target**				
Achieved	490	1709	1.00	
Not Achieved	529	1620	1.21 (1.04 to 1.40)	0.0127
	529	1620	1.20 (1.03 to 1.40)	0.0161
**LDL Target**				
Achieved	2159	7171	1.00	
Not Achieved	687	2269	1.01 (0.91 to 1.12)	0.8776
	687	2269	1.00 (0.90 to 1.12)	0.9419

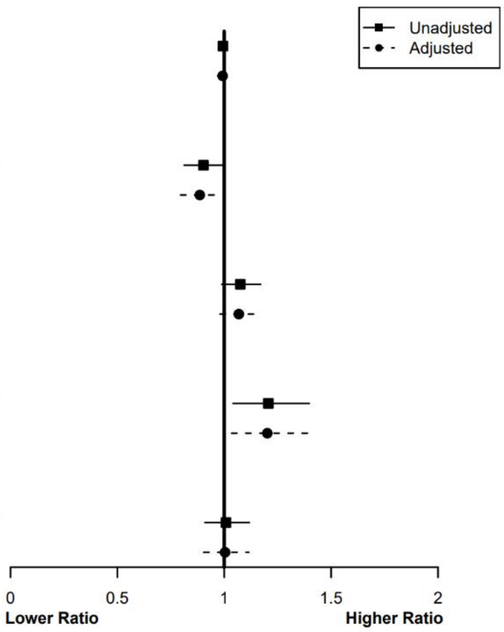

Figure s2: Odds of MIs During Follow Up by Average Statin Adherence and Achievement of Plasma Lipid Targets (from NICE and ESC Recommendations) (Sensitivity inc. incomplete years of follow up)

For peer review only

Table s12: Models for Recurrent MIs by Average Statin Adherence Thresholds (sensitivity inc. incomplete years)

	Continuous Adherence		50% Adherence		80% Adherence	
**Model/Variable**	**Odds Ratio (95% CI)**	**p-value**	**Odds Ratio (95% CI)**	**p-value**	**Odds Ratio (95% CI)**	**p-value**
Unadjusted Model						
**Adherence Target**						
Achieved (ref.)	--		1.00	0.0572	1.00	0.0943
Not Achieved	--		0.90 (0.81, 1.00)		1.08 (0.99, 1.17)	
10% Decrease	0.99 (0.98, 1.01)	0.3392				
Adjusted Model						
**Adherence Target**						
Achieved (ref.)	--		1.00	0.0255	1.00	0.1305
Not Achieved	--		0.89 (0.80, 0.99)		1.07 (0.98, 1.16)	
10% Decrease	1.00 (0.98, 1.00)	0.2279				

For peer review only

STROBE Statement—checklist of items that should be included in reports of observational studies

	Item No	Recommendation	Page No
**Title and abstract**	1	(*a*) Indicate the study's design with a commonly used term in the title or the abstract	1, 2
		(*b*) Provide in the abstract an informative and balanced summary of what was done and what was found	2
**Introduction**			
Background/rationale	2	Explain the scientific background and rationale for the investigation being reported	4
Objectives	3	State specific objectives, including any prespecified hypotheses	4
**Methods**			
Study design	4	Present key elements of study design early in the paper	5
Setting	5	Describe the setting, locations, and relevant dates, including periods of recruitment, exposure, follow-up, and data collection	5
Participants	6	(*a*) *Cohort study*—Give the eligibility criteria, and the sources and methods of selection of participants. Describe methods of follow-up *Case-control study*—Give the eligibility criteria, and the sources and methods of case ascertainment and control selection. Give the rationale for the choice of cases and controls *Cross-sectional study*—Give the eligibility criteria, and the sources and methods of selection of participants	5, Fig 1
		(*b*) *Cohort study*—For matched studies, give matching criteria and number of exposed and unexposed *Case-control study*—For matched studies, give matching criteria and the number of controls per case	N/A
Variables	7	Clearly define all outcomes, exposures, predictors, potential confounders, and effect modifiers. Give diagnostic criteria, if applicable	5-7
Data sources/ measurement	8*	For each variable of interest, give sources of data and details of methods of assessment (measurement). Describe comparability of assessment methods if there is more than one group	5-7
Bias	9	Describe any efforts to address potential sources of bias	7
Study size	10	Explain how the study size was arrived at	5, Fig 1
Quantitative variables	11	Explain how quantitative variables were handled in the analyses. If applicable, describe which groupings were chosen and why	5-7
Statistical methods	12	(*a*) Describe all statistical methods, including those used to control for confounding	7
		(*b*) Describe any methods used to examine subgroups and interactions	7
		(*c*) Explain how missing data were addressed	7
		(*d*) *Cohort study*—If applicable, explain how loss to follow-up was addressed *Case-control study*—If applicable, explain how matching of cases and controls was addressed	N/A

For peer review only

			Cross-sectional study—If applicable, describe analytical methods taking account of sampling strategy	
			(*e*) Describe any sensitivity analyses	7
**Results**				
Participants	13*		(a) Report numbers of individuals at each stage of study—eg numbers potentially eligible, examined for eligibility, confirmed eligible, included in the study, completing follow-up, and analysed	Fig 1
			(b) Give reasons for non-participation at each stage	Fig 1
			(c) Consider use of a flow diagram	Fig 1
Descriptive data	14*		(a) Give characteristics of study participants (eg demographic, clinical, social) and information on exposures and potential confounders	8, Tab 1
			(b) Indicate number of participants with missing data for each variable of interest	Tab 1
			(c) *Cohort study*—Summarise follow-up time (eg, average and total amount)	8
Outcome data	15*		*Cohort study*—Report numbers of outcome events or summary measures over time	Fig 2 Fig 3
			Case-control study—Report numbers in each exposure category, or summary measures of exposure	N/A
			Cross-sectional study—Report numbers of outcome events or summary measures	N/A
Main results	16		(*a*) Give unadjusted estimates and, if applicable, confounder-adjusted estimates and their precision (eg, 95% confidence interval). Make clear which confounders were adjusted for and why they were included	8, 9 Fig 2, Fig 3
			(*b*) Report category boundaries when continuous variables were categorized	6,7
			(*c*) If relevant, consider translating estimates of relative risk into absolute risk for a meaningful time period	N/A
Other analyses	17		Report other analyses done—eg analyses of subgroups and interactions, and sensitivity analyses	8, 9, Supp
**Discussion**				
Key results	18		Summarise key results with reference to study objectives	9
Limitations	19		Discuss limitations of the study, taking into account sources of potential bias or imprecision. Discuss both direction and magnitude of any potential bias	10-12
Interpretation	20		Give a cautious overall interpretation of results considering objectives, limitations, multiplicity of analyses, results from similar studies, and other relevant evidence	10-12
Generalisability	21		Discuss the generalisability (external validity) of the study results	12,13
**Other information**				
Funding	22		Give the source of funding and the role of the funders for the present study and, if applicable, for the original study on which the present article is based	13

*Give information separately for cases and controls in case-control studies and, if applicable, for exposed and unexposed groups in cohort and cross-sectional studies.

Note: An Explanation and Elaboration article discusses each checklist item and gives methodological background and published examples of transparent reporting. The STROBE checklist is best used in conjunction with this article (freely available on the Web sites of PLoS Medicine at http://www.plosmedicine.org/, Annals of Internal Medicine at

For peer review only - http://bmjopen.bmj.com/site/about/guidelines.xhtml

http://www.annals.org/, and Epidemiology at http://www.epidem.com/). Information on the STROBE Initiative is available at www.strobe-statement.org.

For peer review only
